# Predicting future climate at high spatial and temporal resolution

**DOI:** 10.1111/gcb.14876

**Published:** 2019-11-16

**Authors:** Ilya M. D. Maclean

**Affiliations:** ^1^ Environment and Sustainability Institute University of Exeter Penryn UK

**Keywords:** ecology, mechanistic model, microclimate, soil moisture, soil temperature, species distributions

## Abstract

Most studies on the biological effects of future climatic changes rely on seasonally aggregated, coarse‐resolution data. Such data mask spatial and temporal variability in microclimate driven by terrain, wind and vegetation, and ultimately bear little resemblance to the conditions that organisms experience in the wild. Here, I present the methods for providing fine‐grained, hourly and daily estimates of current and future temperature and soil moisture over decadal timescales. Observed climate data and spatially coherent probabilistic projections of daily future weather were disaggregated to hourly and used to drive empirically calibrated physical models of thermal and hydrological microclimates. Mesoclimatic effects (cold‐air drainage, coastal exposure and elevation) were determined from the coarse‐resolution climate surfaces using thin‐plate spline models with coastal exposure and elevation as predictors. Differences between micro and mesoclimate temperatures were determined from terrain, vegetation and ground properties using energy balance equations. Soil moisture was computed in a thin upper layer and an underlying deeper layer, and the exchange of water between these layers was calculated using the van Genuchten equation. Code for processing the data and running the models is provided as a series of R packages. The methods were applied to the Lizard Peninsula, United Kingdom, to provide hourly estimates of temperature (100 m grid resolution over entire area, 1 m for a selected area) for the periods 1983–2017 and 2041–2049. Results indicated that there is a fine‐resolution variability in climatic changes, driven primarily by interactions between landscape features and decadal trends in weather conditions. High‐temporal resolution extremes in conditions under future climate change were predicted to be considerably less novel than the extremes estimated using seasonally aggregated variables. The study highlights the need to more accurately estimate the future climatic conditions experienced by organisms and equips biologists with the means to do so.

## Graphical abstract

Methods for generating fine‐grained, multidecadal estimates of current future temperature and soil moisture are presented. Observed weather data and spatially coherent probabilistic projections of daily future weather were used to drive empirically calibrated physical models of thermal and hydrological microclimates. The models were applied to the Lizard Peninsula, United Kingdom, to provide hourly estimates of temperature at a resolution down to 1 m for the periods 1983–2017 and 2041–2049. High spatio‐temporal resolution extremes in conditions under future climate change were predicted to be considerably less novel than the extremes estimated using coarse‐resolution, seasonally aggregated data.

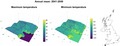

See also the Commentary by https://doi.org/10.1111/gcb.14942.

## 1 INTRODUCTION

Most studies on the climate biology are based on the climatic conditions above‐ground level seasonally averaged across 1 km^2^ or more (Potter, Woods, & Pincebourde, 2013). However, such data fail to capture spatio‐temporal variability in microclimate driven by terrain, wind and vegetation (Bennie, Huntley, Wiltshire, Hill, & Baxter, 2008; Zellweger et al., 2019), and ultimately bear little resemblance to the conditions that organisms experience in the wild (Bramer et al., 2018). This mismatch greatly hinders scientific understanding of the mechanisms explaining how organisms interact with their environment (Kearney & Porter, 2009) and hampers efforts to address the applied challenges such as predicting the ecological consequences of climate change (Potter et al., 2013; Suggitt et al., 2018). Spatial variability in microclimate greatly exceeds the magnitude of climate change expected in the upcoming century, and ignoring this variability leads to erroneous predictions of climate change impacts on species distributions (Gillingham, Huntley, Kunin, & Thomas, 2012; Lembrechts, Lenoir, et al., 2019; Lenoir, Hattab, & Pierre, 2017), population dynamics (Bennie et al., 2013) and behaviour (Blackshaw & Blackshaw, 1994). Failing to account for temporal variability also hinders quantification of exposure to extreme conditions (Parmesan, Root, & Willig, 2000).

To elucidate the mechanistic links between organisms and the climate they experience, and thus provide more robust predictions of biological responses to changing climate under novel conditions, estimates of climate at high spatial and temporal resolution are needed (e.g. Lenoir et al., 2017). For these reasons, there has been a concerted effort to develop efficient and accurate approaches to modelling microclimates, especially in the fields of agriculture and ecology (Bramer et al., 2018). Techniques range from interpolation of in‐situ measurements and statistical downscaling through mechanistic models of physical processes underpinning local climatic variation (Bramer et al., 2018; Lembrechts, Nijs, & Lenoir, 2019). Interpolation methods, while good at capturing temporal variation may fail to capture heterogeneity in microclimate where networks of measurements are sparse (Lembrechts, Lenoir, et al., 2019). Statistical approaches (e.g. Aalto, Harrison, & Lauto, 2017), while excellent at capturing spatial microclimatic variation, are poor at predicting the conditions in novel circumstances (Evans, 2012). Mechanistic methods seek to capture the physical processes driving variation, typically by determining the effects of terrain and vegetation on energy and water fluxes and have been used to reliably capture both spatial and temporal variation (Kearney & Porter, 2017; Maclean, Mosedale, & Bennie, 2019).

One of the earliest mechanistic models used in ecology (Porter, Mitchell, Beckman, & DeWitt, 1973) has been generalized and incorporated into the R package ‘NicheMapR’ (Kearney & Porter, 2017). While tested across a broad range of environments in the context of relatively simple terrain (Kearney et al., 2014), it requires pre‐adjustments of forcing data for important ‘mesoclimate’ effects such as elevation, wind sheltering and cold‐air drainage. It also requires the user to provide estimates of terrain and canopy shading variables. Extending the model of Bennie et al. (2008), Maclean, Suggitt, Wilson, Duffy, and Bennie (2017) developed methods for applying these mesoclimate and terrain adjustments, released as an R package ‘microclima’ (Maclean et al., 2019). However, the ‘microclima’ models must be calibrated with local observations of temperature at the height of interest, whereas ‘NicheMapR’ computes local temperatures from first principles. Elements of both models have subsequently been combined into a single framework, enabling the computation of hourly, historical, terrain‐corrected microclimate anywhere on the earth (Kearney et al., in press). Building on the earlier development of high‐resolution models of soil and surface water conditions (Maclean, Bennie, Scott, & Wilson, 2012), the capabilities of ‘microclima’ have since been extended to enable the estimation of soil water content using the package ‘ecohydrotools’ (Maclean & Mosedale, 2019). All of these models still require hourly or daily weather data to drive them and their application is limited to reconstructing historical microclimates. Never before have these models been used to derive future microclimatic conditions.

While it is inherently impossible to predict the precise climate conditions experienced by an organism at some date and time in the distant future, reliable methods for generating synthetic time series of hourly or daily weather, using weather generators, are increasingly available (Ailliot, Allard, Monbet, & Naveau, 2015; Wilks & Wilby, 1999). Such ‘weather generators’ are capable of reproducing a wide set of climate statistics over a range of temporal scales, from the high‐frequency extremes to the low‐frequency inter‐annual variability for future climate scenarios, as inferred from global climate models (Fatichi, Ivanov, & Caporali, 2011). They thus have a great advantage over other downscaling methods of being able to produce projections on daily or sub‐daily timescales. For the most part, such weather generators can be used for the simulation of weather data at a single site. While in theory it is possible to generate multiple synthetic series for multiple sites, thereby the spatial coherency of the outputs is no longer maintained. This is of limited importance if climate at a given site is unaffected by surrounding conditions, but is of particular relevance in their application in hydrology where lateral flows are important. More recently, therefore, spatially coherent probabilistic estimates of daily weather have been simulated and are available as gridded datasets for specific regions (Met Office Hadley Centre, 2018; Smith, Strong, & Rassoul‐Agha, 2018). Nonetheless, the spatial resolution of such datasets is still relatively coarse (e.g. 12 km, Met Office Hadley Centre, 2018).

Here I demonstrate how spatially coherent probabilistic projections of future daily weather can be coupled to microclimate models to generate hourly simulations of future climate at very high‐spatial resolution. The approach is applied to the Lizard Peninsula, United Kingdom (100 m grid resolution) and Caerthillean Cove on the Lizard Peninsula (1 m grid resolution), to provide hourly estimates of temperature and daily estimates of soil moisture for the 2041–2049 period. These are compared to historic data generated for the 1983–2017 period.

## 2 METHODS

### 2.1 Climate data

The coarse‐resolution data sources used to drive the models over the 1983–2017 period are detailed in Appendix S1. To drive the models over the 2041–2049 period, regional climate model projections produced as part of the UK Climate Projection 2018 (UKCP18) project (Met Office Hadley Centre, 2018; Murphy et al., 2018) were used. This dataset consists of 12 projections from the HadREM3‐GA705 model for RCP8.5 scenario in which emissions are assumed to continue to rise throughout the 21st century. The data are provided as a 12 km gridded dataset of climate variables (maximum and minimum temperature, total incoming shortwave radiation, specific humidity, sea‐level pressure and wind speed) at standard reference height (2 m). Each projection is, in effect, a plausible example of daily weather under global warming. The datasets are spatially coherent and retain physical consistency between the different climate variables. Precipitation was retained as a daily variable. Methods used to disaggregate remaining climate variables, both observed to projected, to hourly are detailed in Appendix S1.

At high‐temporal resolution, many of the differences in extreme values between historic and projected climate may be due to systematic differences in the datasets caused by scaling effects and methodological assumptions inherent in the HadREM3‐GA705 model. To enable such biases to be corrected, each model projection also covers historic periods, enabling direct comparison with climate observations. The 12 climate projections for the 2000–2010 period were thus downloaded, converted to hourly and the frequency distribution of each climate variable compared to that of observed data. Both datasets were then ranked, and following the exploratory analyses to establish the required sample size to adequately represent the frequency distribution of data, a series of 1,200 equal‐interval values, spanning the full range of values in both datasets, were randomly selected. Generalized additive models were then fitted to define the mathematical relationships between observed and modelled data, and the same transformations then applied to the 2041–2049 datasets. Code for performing these adjustments and for deriving sea‐surface temperature under future climate have been bundled into the R package ‘UKCP18adjust’. The package is available on Github (ilyamaclean/UKCP18adjust). Further details are provided in Appendix S1.

### 2.2 Downscaling climate data

Using the R package ‘microclima’ (Maclean et al., 2019), mesoclimate effects were determined by fitting thin‐plate spline models to hourly differences between land and sea temperature data with elevation, coastal exposure upwind and mean coastal exposure in all directions included as covariates. The thin‐plate models were then applied to derive land–sea temperature differentials for specific locations at high resolution using higher resolution versions of the same predictor variables.

Following Bennie et al. (2008) and Maclean et al. (2019), near ground‐surface microclimate temperatures were modelled using an energy balance equation in which the difference between microclimatic and mesoclimatic reference temperature at 2 m is modelled as a function of energy fluxes occurring at the surface—net radiation, latent heat, energy fluxes to/from the soil and a resistance to the loss of sensible heat. Assuming that latent heat and soil fluxes are small and proportional to net radiation, the temperature difference is a linear function of net radiation, and the gradient of this relationship is a measure of the thermal coupling of the surface to the atmosphere. The gradient varies as a function of both the structure of the vegetation, the height above the ground for which microclimate temperature estimates are required, and wind speed, and was fitted using field calibration data. In the 1‐m resolution model, the effects of canopy shading on radiation and vegetation on near‐surface wind speeds were accounted for, by computing surface roughness and the topographic shelter coefficient of Ryan (1977). In the 100‐m resolution model, radiation and wind speed were downscaled by accounting for the local terrain and by assuming a standard logarithmic wind‐height profile for a grass surface (Allen, Pereira, Raes, & Smith, 1998). Further methodological detail is provided in Maclean et al. (2019). To downscale the precipitation, it was necessary to account for both elevation‐driven variation in total rainfall, and variation in the number of rainfall days. For historic data, and future projections, thin‐plate spline models were fitted to these data with elevation as a covariate. The thin‐plate models were then applied at 100 m resolution to derive downscaled estimates for the Lizard Peninsula. Further details are provided in Appendix S1.

### 2.3 Soil moisture

Daily high‐resolution soil moisture estimates were derived using the R package ‘ecohydrotools’ (Maclean & Mosedale, 2019). This package implements a modified spatial version of the Mahrt and Pan (1984) two‐layer model of soil hydrology. In each delineated hydrological basin, fractional soil water content is computed in a thin upper layer for use in calculation of bare soil evaporation. Water storage is computed for an underlying deeper layer. Precipitation enters the top soil layer, but any precipitation that cannot infiltrate or re‐evaporate is specified as runoff. Evapotranspiration from vegetated portions of the surface was partitioned equally between both layers. Evapotranspiration was calculated using ‘ecohydrotools’ in which daily evapotranspiration is calculated from hourly meteorological data using the FAO Penman–Monteith method (Allen et al., 1998). The input meteorological data were the downscaled, terrain‐adjusted values provided by ‘microclima’. Runoff rates were calculated using the curve number method (Mishra & Singh, 2013). Using this approach, run‐off varies as a function of precipitation and the soil infiltration capacity, itself dependent on soil properties, land cover and the hydrological condition of the soil. Using ‘ecohydrotools’, the rate and direction of exchange of water between the soil layers is determined by the hydraulic diffusivity and conductivity and by the difference in soil moisture between the two layers. Hydraulic diffusivity and conductivity are determined from antecedent soil moisture and five parameters describing the hydraulic properties of the soil (Table S1), using soil‐water retention equations described by Van Genuchten (1980). Bare soil evaporation was confined to the top soil layer, whereas evapotranspiration from vegetation areas was equally apportioned between both layers. Within each time‐step, soil and surface water were spatially distributed across the basin by the Bevan and Kirkby (1979) topographic wetness index. Surplus surface water remains within the basin unless the basin volume is exceeded, in which case it is accrued to the adjoining basin at the pour point. Consequently, the model was run iteratively for each basin starting with the basin with the highest elevation pour point.

### 2.4 Application and validation of the model

**Figure 1:**
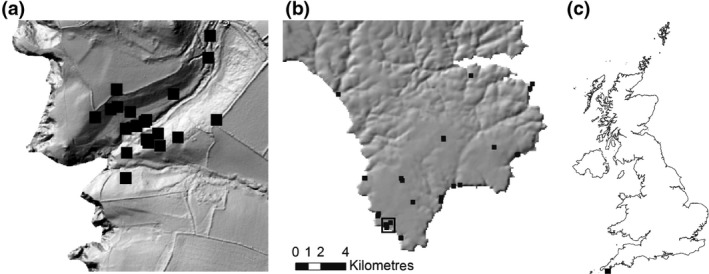
Study areas depicting the locations covered by the microclimate (a) and mesoclimate (b) models in the south‐west of the United Kingdom (c). Black squares indicate the locations of iButton temperature data loggers

The models were applied at grid resolution of 100 m over the Lizard Peninsula (50°20N, 5°100W) and at 1 m resolution over part of the Lizard Peninsula in Caerthillean Cove (Figure 1). Gridded temperature datasets were derived for 5 cm above‐ground level and across the entire Lizard provided as two datasets: one for open ground with no canopy shading, in which microclimate temperatures are influenced strongly by the radiation and the second for closed canopy, in which microclimate temperatures are minimally influenced by the radiation. In Caerthillean Cove, spatial variation in canopy‐shading effects were estimated from aerial photographs and LiDAR data but, in the absence of an available time‐series, were assumed to be time‐invariant (see Maclean et al., 2019). Across the entire Lizard Peninsula, in the absence of available information, constant soil properties were assumed (Table S1). No adjustments were made for vegetation type in the calculation of evapotranspiration, and the bare soil fraction was assumed constant at 0.2, a value typical of the study region (Maclean, Hopkins, Bennie, Lawson, & Wilson, 2015). The provided soil moisture estimates are for 0–10 cm depth, obtained by averaging daily moisture across both soil layers.

The temperature models were calibrated and tested by comparing the model predictions with the observed data obtained from 56 iButton thermochrons deployed 5 cm above ground across study sites over the March 2010–December 2014 period (see also Maclean et al., 2017, 2019). Half the data were used for calibration and the other half for validation. Data were partitioned by time period and location to ensure whether the validation data were independent of calibration data. The soil moisture models were tested by comparing model predictions to 10,000 field measurements of soil moisture obtained from 250 locations distributed widely across the study site between April 2010 and March 2011 (see also Maclean et al., 2012). No calibration is necessary in the soil moisture model as estimates are derived entirely from first principles.

### 2.5 Bioclimate variables

To examine the climatic changes, the 19 bioclimate variables available from Wordclim that are commonly used for species distribution modelling (Fick & Hijmans, 2017) were calculated (Table 1). Additionally, 11 climatic variables, recognized as being physiologically important for species, but not included in the Wordclim dataset (Gardner, Maclean, & Gaston, 2019), were computed (Table 1). Each variable was calculated for each year and each model run of future climate. Additionally, means over the periods 1983–2017 and 2041–2049 and decadal trends in each variable, derived using linear regression on annual values, were computed. Finally, to assess novelty in climate, a novelty index was computed separately for each grid cell, representing the proportional overlap in the frequency distributions of annual values in the historic period with those for all model runs in the future period (0 = complete overlap, 1 = no overlap).

**Table 1:** Bioclimate variables calculated to determine change

Variable	Descriptor
BIO1	Mean annual temperature (°C)
BIO2	Mean diurnal range (°C)
BIO3	Isothermality (BIO2/BIO7) × 100
BIO4	Temperature seasonality (°C standard deviation × 100)
BIO5	Maximum temperature (°C)
BIO6	Minimum temperature (°C)
BIO7	Temperature annual range (°C, BIO5 − BIO6)
BIO8	Mean temperature of wettest quarter (°C)
BIO9	Mean temperature of driest quarter (°C)
BIO10	Mean temperature of warmest quarter (°C)
BIO11	Mean temperature of coldest quarter (°C)
BIO12	Annual precipitation (mm)
BIO13	Precipitation of wettest month (mm)
BIO14	Precipitation of driest month (mm)
BIO15	Precipitation seasonality (mm coefficient of variation)
BIO16	Precipitation of wettest quarter (mm)
BIO17	Precipitation of driest quarter (mm)
BIO18	Precipitation of warmest quarter (mm)
BIO19	Precipitation of coldest quarter (mm)
PHYS1	Mean fractional soil water content during growing season^a^
PHYS2	Mean growing season^a^ temperature (°C)
PHYS3	Total precipitation during growing season^a^ (mm)
PHYS4	Length of growing season^a^ (days)
PHYS5	Mean Jun–Aug fractional soil water content
PHYS6	Frost hours
PHYS7	Frost‐free season length (days)
PHYS8	Hours with temperature >25°C
PHYS9	Consecutive days when soil is water‐logged
PHYS10	Consecutive days with soil moisture at wilting point
PHYS11	Growing degree‐hours/1,000

a Growing season defined as period where 5‐day means of precipitation exceeds half the potential evapotranspiration and temperatures lie between 5 and 35°C.

## 3 RESULTS

The mesoclimate temperature model had a mean absolute error (MAE) of 0.97°C and root mean square error (RMSE) of 1.23°C, whereas the microclimate temperature model had an MAE of 1.25°C and RMSE of 1.61°C. The soil moisture model predicted fractional soil moisture with an MAE of 0.013 and an RMSE of 0.020 (Figure S5).

**Figure 2:**
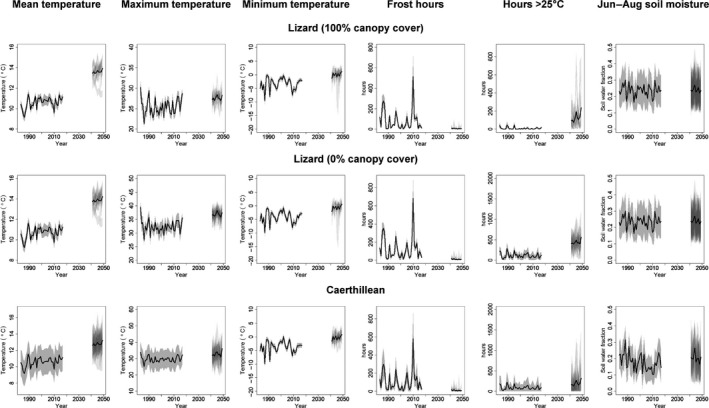
Trends in selected bioclimate variables. Black lines show the mean value across the study period and, in 2041–2049 across model runs in each year. Grey shading in the period 1983–2017 represents ±2 *SD* in the spatial variability. In the period 2041–2049, semi‐transparent shading is used to depict ±2 *SD* in spatial variability of each model run and darker shading thus indicates greater overlap between model runs. More detailed variable descriptors are provided in Table 1. Trend plots for all variables are in Supplementary Results

**Figure 3:**
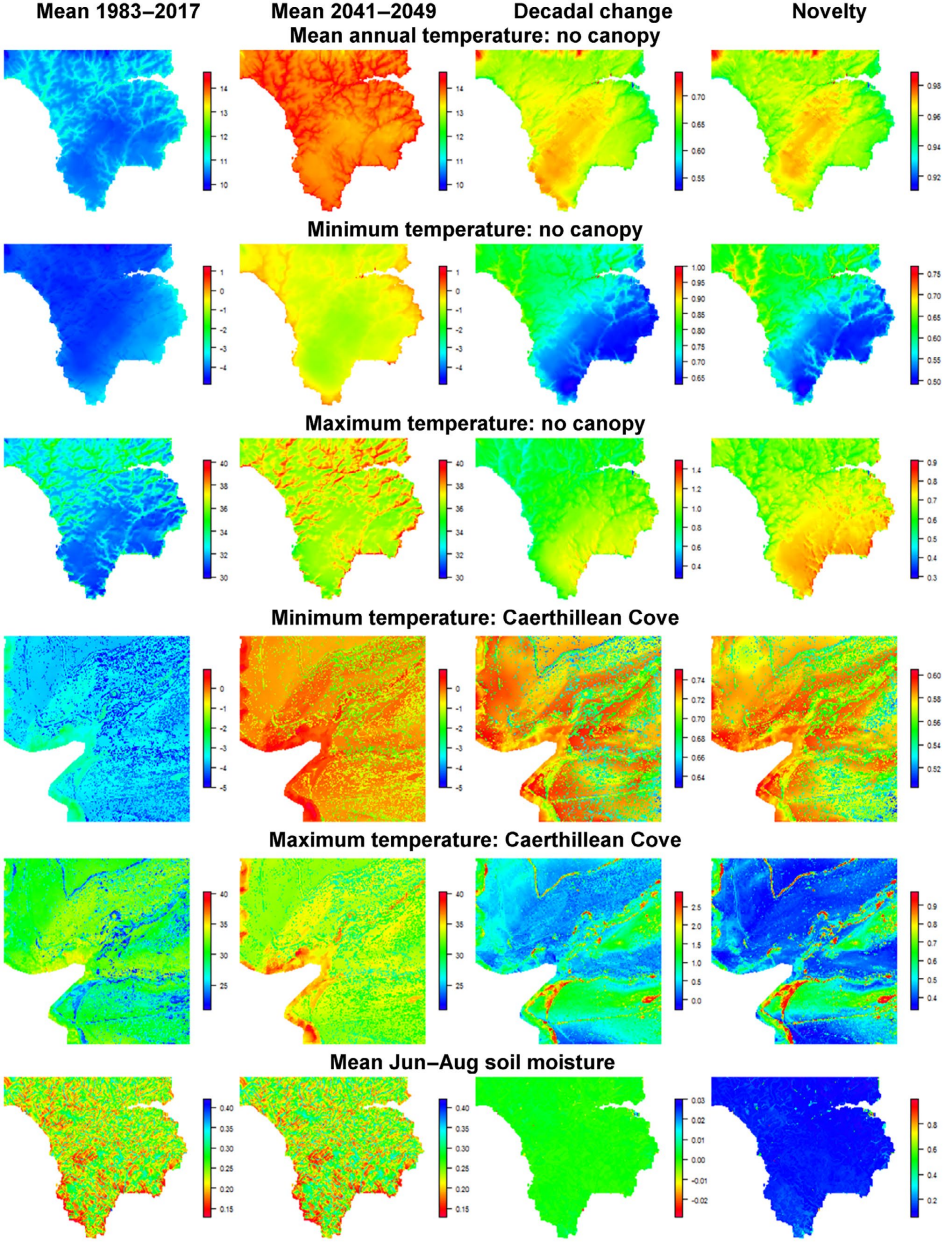
Maps of selected bioclimate variables. Decadal changes were derived using linear regression on yearly values. Novelty represents the proportional overlap in the frequency distribution of annual values in 1983–2017 with that of annual values for each model run in 2041–2049 (0 = complete overlap, 1 = no overlap)

Despite exhibiting moderate inter‐annual variability, mean annual temperatures increased throughout the study period, exhibiting a 0.54–0.71°C decadal increase across the Lizard under closed canopy, a 0.52–0.74°C decadal increase under open canopy and a 0.34–0.72°C increase in Caerthillean Cove (Figure 2, left). Conditions in 2041–2049 across the Lizard were almost entirely novel relative to the 1983–2017 baseline period (novelty index range closed canopy: 0.92–0.98; open canopy: 0.91–0.99), though were predicted to be marginally less so in Caerthillean (novelty index range: 0.74–0.96; Figure 3, right). There was some evidence of acceleration in warming, predicted to be greatest at higher elevations and lowest on north‐east‐facing coastlines and in Caerthillean, under closed canopy (Figure 2, left; Figure 3, top). Increases in temperature were generally predicted to be greatest during the warmest quarter of the year, exhibiting a 0.7–0.9°C decadal increase under closed canopy, 0.6–0.9°C decadal increase under open canopy and 0.38–1.1°C decadal increase in Caerthillean. In contrast, during the coldest quarter, temperatures were predicted to increase by 0.45–0.66°C per decade under closed canopy, by 0.50–0.75°C per decade under open canopy and by 0.44–0.57°C per decade in Caerthillean (Figures S6–S11).

Maximum temperatures exhibited high inter‐annual variability and changes in this varied widely across the study region, ranging from a 0.1°C per decade decrease on south‐facing slopes in Caerthillean Cove to a 2.8°C increase under dense canopy in sheltered valleys (Figure 3). Changes in minimum temperatures were predicted to be more consistent spatially, but exhibited a high degree of inter‐annual variability (Figure 2, middle). Decadal changes ranged only from 0.63 to 0.96°C, and were also greatest under dense canopy in sheltered valleys (Figure 3). Compared to seasonal and annual temperatures, the novelty of conditions of minimum temperatures in 2041–2049 relative to 1983–2017 was generally lower (Figures S6–S11). Index values ranged from 0.49 under open canopy at exposed sites in the south‐east of the Lizard to 0.74 in sheltered valleys of the north‐west. The novelty of maximum temperatures varied greatly, with index values ranging from 0.2 in coastal regions of the north to 0.98 under closed canopy in Caerthillean (Figure 3).

Mean annual precipitation was predicted to increase across the study region, though there was relatively high variance among model runs for future predictions (Figures S6–S8). Nevertheless, decadal changes were predicted to be in the order of 30–120 mm per decade and greatest in the relatively drier south‐east coastal region of the Lizard (Figures S9–S11). Across the entire study region, the changes were predicted to be most pronounced during the driest month where precipitation was predicted to become almost twice as high (Figure S6). There was a marked predicted decrease in the seasonality of precipitation, such that conditions in 2041–2049 relative to 1983–2017 were almost entirely novel (novelty index range: 0.92–0.97; Figures S9–S11). Soil moisture exhibited a high spatial and inter‐annual variability, but little consistent trend through time (Figure 2, right; Figures S6–S8). Trend plots and maps depicting the 1983–2017 and 2041–2049 means, decadal changes and novelty indices of every variable are provided in Appendix S2.

## 4 DISCUSSION

The purpose of this study was to demonstrate the potential to model future climatic conditions at high spatial and temporal resolution. While it is not possible to test how well the model performs under future conditions, the predictive capacity of the model was high, explaining over 90% of the variation in soil moisture and local temperature anomalies over the period in which validation was carried out. The performance of the microclimate models over historic periods is discussed in detail in Maclean et al. (2012, 2017, 2019) and discussion here is limited to its likely performance under future conditions, except to acknowledge the limitation imposed by assuming time‐invariant canopy‐cover and uniform soil properties. The dominant vegetation types are perennial grasses *Ulex* spp., which are not especially prone to seasonal changes. Nonetheless, dense stands of *Salix caprea*, *Prunus spinosa* and *Rubus* spp. are present in valley bottoms, and failure to account for seasonal variation in cover remains a limitation of this study. The assumption of spatially uniform soil properties, particularly soil depth, is also problematic, though somewhat offset by assuming a relatively deep underlying soil layer (Mahrt & Pan, 1984). Nevertheless, in areas with shallow soil, seasonal fluctuations in moisture are likely to be underestimated.

The probabilistic regional projections used to drive the microclimate models are derived from climate models, which approximate the real climate system, and there are known systematic differences between climate model results and observations (Murphy et al., 2018). Of particular note, both solar radiation and the proportion of days with zero precipitation are underestimated substantially in the model projections. Some of the regional patterns caused by terrain and coastal effects are poorly represented. Although largely corrected by applying adjustments to the data, there remains the possibility that the modelled future changes in climate are partially an artefact of biases in climate projections. Biases in extreme values are particularly problematic to correct (Christensen, Boberg, Christensen, & Lucas‐Picher, 2008), and estimates of maximum and minimum temperature changes should thus be treated with caution. In addition, though the 12 realizations of climate projections cover a broad range of potential future climate pathways, some potential influences on future climate are not yet fully understood. It is possible, therefore, that real‐world future changes will lie outside the envelope of the estimates presented here.

Apart from these limitations, the present study provides a promising means of deriving future climatic conditions at high spatial and temporal resolution. Many existing studies on climate change impacts neglect the most important biophysiological variables, which typically reflect proximal exposure to conditions that affect performance or the timing of climate events in relation to circannual rhythms (Gardner et al., 2019). Deriving these variables is only possible with high spatial and temporal resolution climate data (Gardner et al., 2019; Kearney & Porter, 2009). Although coarse‐resolution climate data are assumed to be statistical meaningful predictors of biological responses through ‘mean field approximation’ (Bennie, Wilson, Maclean, & Suggitt, 2014), unquantified additional factors may partially drive the apparent relationship with climate (Dormann et al., 2012). The influence of these additional variables may vary in new locations or over new time periods and thus lead to unreliable predictions (Austin, 2002). Basing future predictions purely on changes to coarse‐resolution climate may therefore be problematic if the climatic component of the original correlation does not match physiologically relevant patterns of variation.

It should be acknowledged, however, that the approach presented here is only feasible over relatively small regions. Accurate representation of global or regional climate at high spatio‐temporal resolution is impractical, even with rapid advances in computer processing power and high‐resolution remote sensing data. However, the methods presented in this manuscript potentially facilitate the identification of the conditions under which mean field approximations break down, and the spatial scales at which this breakdown occurs. Such breakdowns are likely when mean climate conditions are not closely correlated with exposure to conditions that affect the performance and survival of organisms as may occur, for example, when microclimate heterogeneity is high (Suggitt et al., 2018) or when organisms exhibit thermoregulatory behaviour (Kearney, Shine, & Porter, 2009). The methods presented thus strengthen the ability to provide general recommendations for the appropriate spatial and temporal scales at which best to model the responses of species to climate change, complementing recommendations in other studies (e.g. Lenoir et al., 2017).

The results indicate that the degree of spatial covariance between extreme conditions measured using hourly data and those derived from seasonally aggregated data is relatively low, particularly at higher resolutions. In consequence, the extent to which low temporal resolution data adequately capture physiologically meaningful exposure to climatic conditions is questionable. For example, at fine resolution, maximum temperatures are influenced strongly by solar radiation and are hence highest on south‐facing slopes, whereas mean summer temperatures follow a pattern that is largely altitude‐dependent. The novelty of conditions is also lower for extreme conditions than it is for seasonal averages, implying that studies using seasonally aggregated data may overestimate the impact of climate change. The degree of covariance may be much stronger over regional and global scales, where differences in temperature and evapotranspiration are primarily latitude‐dependent (Fick & Hijmans, 2017). However, the fine‐resolution spatial differences in climate predicted by this study, particularly in extreme conditions, are nearly as large as coarse‐resolution differences over entire continents. Across the four hectare region Caerthillean Cove, for example, maximum temperatures varied by almost 20°C. Climate variables derived using coarse‐resolution data may thus bear little resemblance to conditions experienced by organisms, which at worst may yield highly erroneous predictions, and at best will greatly increase uncertainty (e.g. Lembrechts, Lenoir, et al., 2019; Suggitt et al., 2018).

It is hoped that this study encourages biologists to consider future climatic changes at finer spatial and temporal resolution, as in doing so they will make more robust predictions. I am confident that the approach proposed here can be applied to other locations and could improve understanding of biological responses to climate change.

## Data Availability

The data that support the findings of this study are available from the Met Office. Restrictions apply to the availability of these data, which were used under license for this study. Data are available from https://catalogue.ceda.ac.uk with the permission of the Met Office.
