# Supplementary material for: Predicting future climate at high spatial and temporal resolution
Source: Glob Chang Biol. 2019 Nov 16;26(2):1003–11. doi: 10.1111/gcb.14876 (PMC7027457; doi:10.1111/gcb.14876)
Supplement: Supplementary file 1 [file GCB-26-1003-s001.pdf]

SUPPLEMENTARY METHODS

Climate data sources

To drive the models over the period 1983-2017 the following coarse-resolution historic climate datasets were used: (1) daily precipitation and minimum and maximum temperature, available at one km grid resolution from the UK Met Office (Met Office, 2018), (2) six-hourly sea-level pressure, wind speed and direction and specific humidity available at ~1.9° grid resolution from the National Weather Surface National Centres for Environmental Prediction (NOAA-NCEP, Kanamitsu et al., 2002), (3) hourly surface incoming shortwave radiation available at 0.05° grid resolution from the EUMETSAT Satellite Application Facility on Climate Monitoring (CMSAF; Posselt, Müller, Trentmann, Stockli, & Liniger, 2014) and (4) daily mean sea-surface temperatures available at 0.25° grid resolution from the National Oceanic and Atmospheric Administration (NOAA; Reynolds et al., 2007). Six-hourly humidity and pressure data and daily sea-surface temperature data were interpolated to hourly using the native spline function of R (R Core Team, 2018). Wind speed and direction were used to derive easterly and northerly wind vectors, which were then spline-interpolated to hourly before back-calculating hourly wind speeds and directions. Hourly temperature data were derived from daily maxima and minima using the 'hourlytemp' function in 'microclima' whereby diurnal patterns and variation in cloud cover and radiation are accounted for (see Maclean et al., 2019 for further details). Total incoming shortwave radiation was partitioned into its direct and diffuse components using the approach outlined by Skartveit et al., (1998) implemented by the 'microclima' function 'difprop'. An index of cloud cover was derived from radiation data by computing the proportion of actual to clear-sky radiation, with values at night derived using spline interpolation.

To drive the models over the period 2041-2049, regional climate model projections produced as part of the UK Climate Projection 2018 were used (Met Office Hadley Centre, 2018). The UKCP18 datasets consists of 3600 daily values for the period 1st December 2040 to 30th Nov 2050, which in fact spans 3652 days. Values for the 31st of December, May, July, August and October and the 29th of February in leap years were therefore omitted and the 3600 daily values matched to the remaining dates. Values for the omitted dates were then derived using spline interpolation. Daily data, with the exception of radiation and rainfall (see below), were interpolated to hourly using the native spline function of R (R Core Team, 2018).

Deriving hourly diffuse and direct radiation for future climate scenarios

The shortwave radiation in the UKCP dataset is daily, but for coupling with microclimate models, it is necessary to derive an hourly dataset. Incoming shortwave radiation is affected by the optical depth but is also dependent on the optical path length, which follows a predictable diurnal cycle. Since the optical depth is primarily affected by cloud conditions, but is unaffected by solar altitude, it does not follow a predictable diurnal cycle. It is therefore more appropriate to interpolate this variable to hourly. Optical depth (o) was derived as follows:

$$o = \frac{log(R/R_0)}{\overline{m}^N}$$

Here, R is incoming shortwave radiation, R_o is the solar constant (~4.87 MJ m^{-2} hr^{-1}), $\overline{m}$ is the mean of the airmass coefficient (Kasten & Young, 1989) computed by deriving the inverse at hourly intervals for a 24 hour period corresponding to each day, and N is a power adjustment to the airmass coefficient, to account for the reduced effect of the airmass coefficient on radiation transmission under cloudy conditions. N is given by:

$$N = exp(0.783 - \ 0.436log_e(\bar{o})$$

where $\bar{o}$ is the assumed optical depth when N = 1. This relationship was empirically derived using radiation data obtained from Camborne weather station (50.2178°N, -5.32656°W) for the period 1995-2000. The resulting optical depth values were then interpolated to hourly, and hourly radiation calculated by inverting calculations above as follows:

$$R = R_o \exp(-m_h{}^N)$$

Where m_h is the hourly airmass coefficient. Airmass and clear sky coefficients were derived using functions available in the R package ‘microclima’ (Maclean, Mosedale, & Bennie, 2019). The result is a diurnal cycle in radiation superimposed over the daily values (Fig S1).

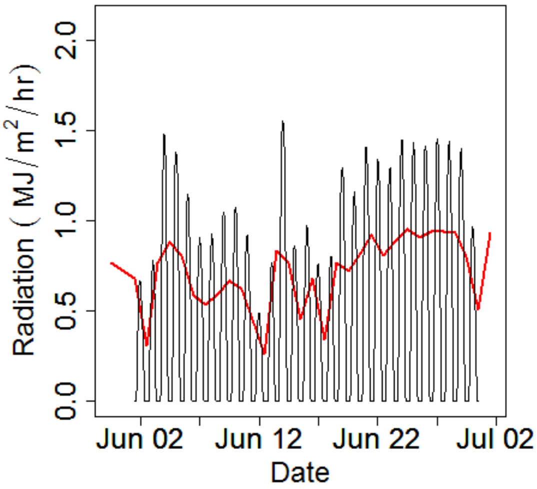

Fig S1. Hourly radiation derived from daily values. Example from simulation 1, June 2041.

Additionally, since the original 12 km data were resampled from 60 km resolution data, and optical depths spline interpolated, spatial and temporal patchiness in cloud cover and hence direct radiation is underestimated. We therefore simulated spatially and temporally autocorrelated patchiness in optical depth. To do so, we estimated the degree of patchiness and spatio-temporal autocorrelation in this from observed data for Cornwall in 2005. We first computed six-hourly deviations in optical depth from daily means and hourly deviations from six-hourly data and fitted spatial variogram models using the R package ‘gstat’ (Pebesma & Graeler, 2019). We then used the parameters from these variogram models to simulate spatially-autocorrelated grids of six-hourly and hourly deviations from daily data using the ‘predict’ function in ‘gstat’ and added these gridded datasets to the real gridded data for each hour. The net result is a gridded dataset that preserves the main spatial trends in optical depths but simulates artificial and temporally-autocorrelated patchiness in optical depth consistent with patchiness in real data (Fig. S2). Code for interpolating daily data to hourly and introducing patchiness are included in the package ‘UKCP18adjust’.

Downscaling rainfall data

For historic data (one km grid resolution), monthly precipitation and the number of precipitation days in each month were computed for 2,300 km^2 of west Cornwall (49.82 to 50.38ºN, 5.72 to 4.85ºW) and thin-plate spline models fitted to these data with elevation as a covariate. The thin-plate models were then applied at 100 m resolution to derive downscaled estimates for the Lizard Peninsula. The following adjustments were then performed to derive daily rainfall estimates within each month. First, one km resolution data were spatially interpolated to 100 m grid resolution. This smooths the data spatially, but fails to account for elevation effects. Thus, where the resulting number of precipitation days exceeded the (elevation-adjusted) modelled monthly number, the requisite number of days with the lowest precipitation were set

to zero. Where the modelled monthly number of precipitation days exceeded the spatially derived total, the required number of days with zero rainfall was replaced with randomly generated precipitation data, with random data drawn from a statistical distribution consistent with actual precipitation data. The zero precipitation days selected for replacement were those judged most likely to receive precipitation based on summed precipitation across the whole of west Cornwall. The UKCP18 datasets (12 km grid resolution) were downscaled in identical ways, except that the whole of the UK was used to fit the thin-plate spline models to accommodate the coarser resolution at which these data are available.

Climate dataset adjustment

To correct for differences in the range and frequency distribution of values in the historic climate and UKCP18 datasets, we applied a correction function as follows. The parameters for applying this correction function were derived by obtaining historic observed data and UKCP18 data for the period 2001-2009, interpolating the UKCP18 dataset to 5 km grid resolution to match historic data, and extracting the data for Cornwall and the Isles of Scilly, UK from both datasets. Both datasets were then ranked and 10,000 equally spaced values spanning the full range of values in both datasets randomly selected. Coefficients were then derived by applying General Additive Models using the 'gam' function for R (R Core Team, 2018). The correction functions were then applied to all UKCP18 data for the period 2041-2049. An example of the correction, for net shortwave radiation, is show in Fig S3.

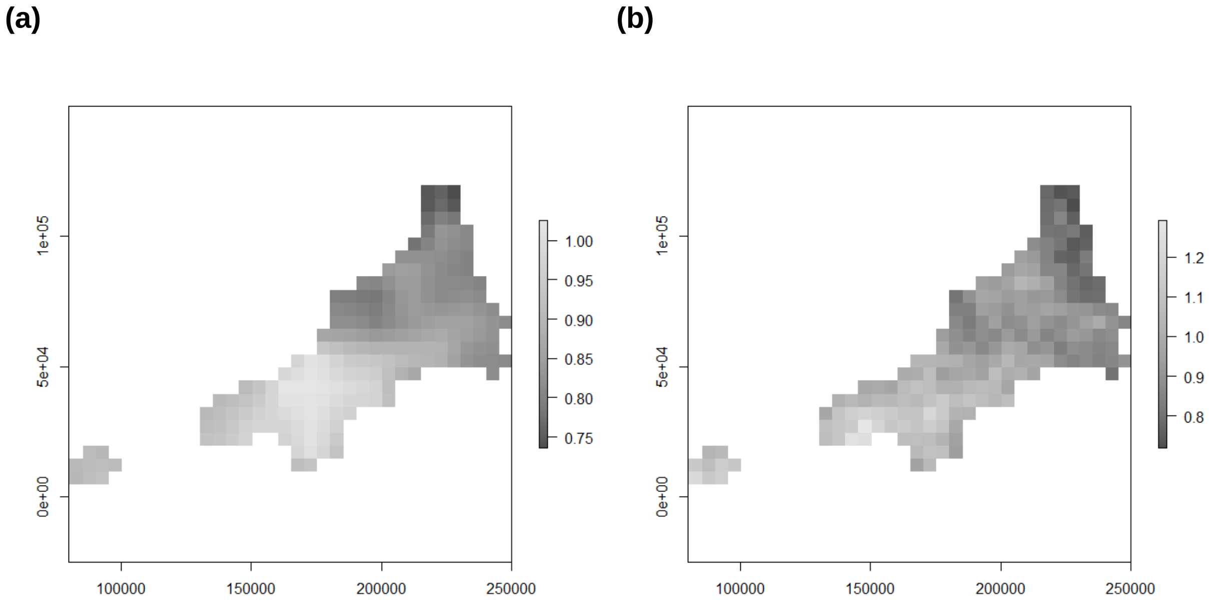

Fig S2. Spatial variation in optical depth (11:00 1st Dec 2000) in the UKCP18 model run 8 across Cornwall prior to simulating patchiness (a) and after simulating patchiness (b)

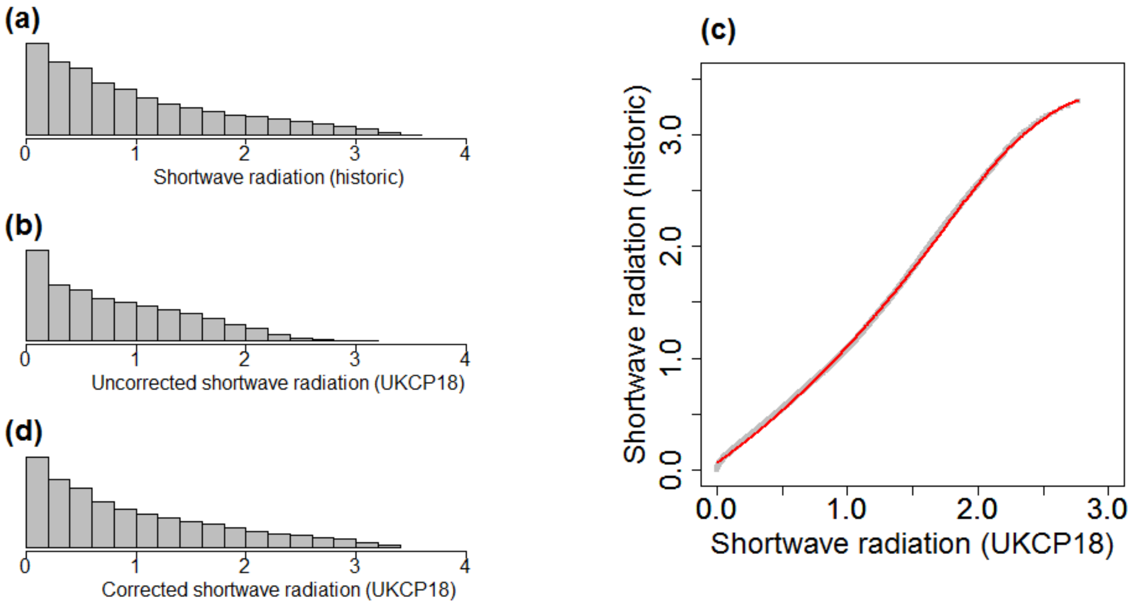

Fig S3. Frequency distributions of hourly non-zero shortwave radiation across Cornwall, UK (MJ m^{-2} hr^{-1}) in the observed historic (a) and UKCP18 datasets (b). In (c) 1200 data points from each dataset, spanning the full range of non-zero values, are plotted against one another (grey dots), and used to derive correction functions (red line). The effects of applying these correction functions on the frequency distribution of shortwave radiation values in the UKCP18 dataset are shown in (d).

No adjustment was required for mean daily temperature, but similar adjustments were applied to diurnal temperature ranges, wind speed, sea-level pressure and specific humidity. We also applied elevation corrections to temperature (*T*) and pressure (*P*) as follows:

$$T = \bar{T} + l\bar{z}$$

$$P = P_s \frac{(293 - 0.0065z)^{5.26}}{293}$$

Where $\bar{T}$ is uncorrected temperature, $\bar{z}$ is the elevation differences between 5 km gridded data and the 60 km gridded data from whence the 12 km UKCP18 data were originally derived and *l* is the lapse rate calculated using the 'lapserate' function in 'microclima'. P_s is sea level pressure in Pascals and *z* is elevation in metres.

Precipitation was retained as daily data. Prior to applying corrections we applied elevation corrections. We first coarsened the data to the original 60 km resolution and computed total precipitation amount and the proportion of precipitation days in each grid cell. We then fitted thin-plate spline models with 60 km gridded elevation as a covariate and then applied the same spline models to Cornwall using five km gridded elevation data to provide higher-resolution elevation adjusted estimates of total precipitation and the number of precipitation days. We then resampled each daily estimate to five km resolution using bilinear interpolation and set the number of zero precipitation days to be consistent with elevation derived predictions as follows. Where predicted days with zero precipitation exceeded the total in interpolated data, the requisite number of days with the lowest precipitation were set to zero. Where days with zero precipitation in interpolated data exceeded the predicted total, we generated a random dataset of precipitation with a statistical distribution consistent with actual precipitation data, and replaced zero precipitation in the interpolated data set with low-ranking precipitation amounts in the randomly generated dataset on days judged most likely to receive precipitation based on precipitation patterns across the whole of Cornwall and the UK

generally. For each grid cell, we then computed the total precipitation amount and applied a constant multiplier to ensure consistency with the estimated elevation-adjusted total. The same approach, based on applying general additive models, was used to ensure consistency with observed data, except that (1) the ratio of observed to UKCP18-modelled days with zero precipitation was computed and applied to UKCP18 data to increase the total number of days with zero precipitation ~8-fold. (2) Non-zero data were log-transformed prior to applying model corrections and then back-transformed after correction. Functions and code for applying these corrections are included in the package 'UKCP18adjust'.

Sea-surface temperature

Though evidently included in the General Circulation Model to derive land surface temperatures, projected high temporal-resolution sea-surface temperature data were unavailable for download. Consequently, we derived an empirical relationship to obtain sea-surface temperatures from land temperatures:

$$SST_t = SST_{t-1} + \alpha_1 \Delta T_1 + \alpha_2 \Delta T_2 + k$$

where k is a constant, $\Delta T_1$is the difference between land and sea surface temperatures given by:

$$\Delta T_1 = T_t - SST_{t-1}$$

where T is land temperature. ΔT_2 is the difference between sea-surface temperature and the sea temperature at greater depth, and is approximated as follows:

$$\Delta T_2 = SST_{t-1} - \bar{T}$$

where $\bar{T}$ is mean annual temperature. The coefficients α_1 defines the strength of thermal coupling between the sea surface and the atmosphere and α_2 the strength of thermal coupling between the sea surface and underlying sea layer. The parameter k defines the warming effect due to North Atlantic drift. The parameters α_1, α_2 and k were derived using the historic datasets averaged across Cornwall through a process of iterative fitting (Fig S4a). Projected sea-level temperatures for all 12 models runs in each year (2041-2049) are shown in Fig S4b.

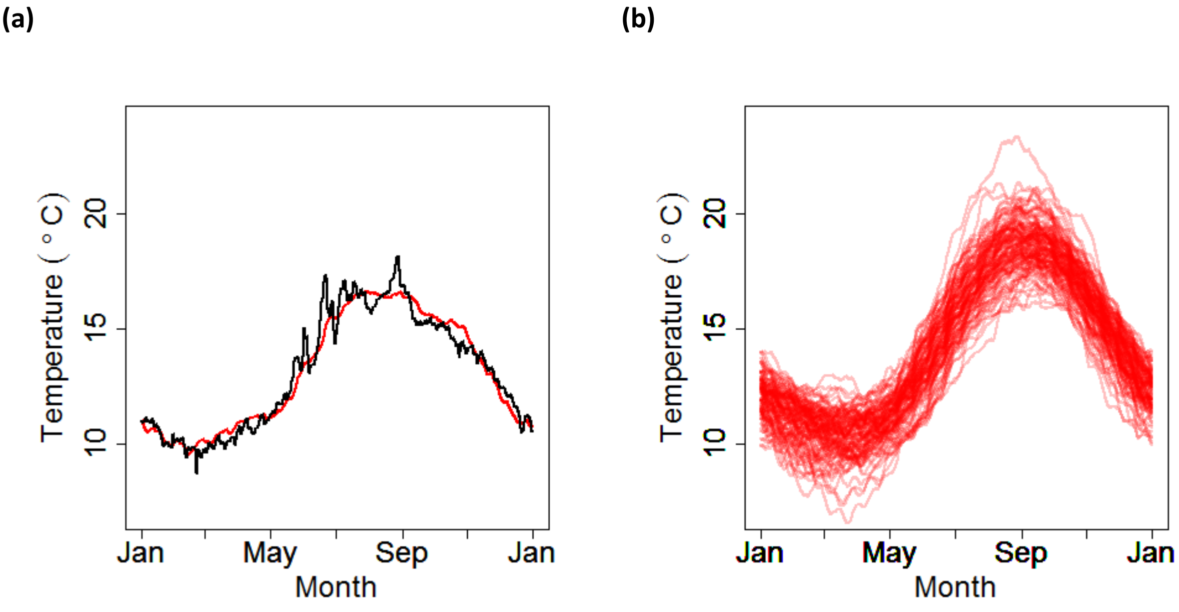

Fig S4. In (a) modelled (red) and observed (black) sea-surface temperatures in 2017 are shown. In (b) modelled sea-surface temperatures for all 12 model runs and years (2041-2049) are shown.

Table S1. Parameters used to run the hydrological model.

Parameter	Description	Value
n	Pore size distribution parameter (dimensionless)	1.35
α	Van Genuchten shape parameter (cm^{-1})	0.0213
K_{Sat}	Saturated hydraulic conductivity (cm / day)	5.89
Θ_{min}	Residual water content (cm^3/ cm^3)	0.091
Θ_{max}	Water content at saturation (cm^3/ cm^3)	0.419
Θ_1	Initial soil water fraction of the surface layer	0.35
Θ_2	Initial soil water fraction of sub-surface layer	0.35
d_0	Initial surface water depth (mm)	0
p_1	Power adjustment applied to topographic wetness index values for surface layer	0.2
p_2	Power adjustment applied to topographic wetness index values for sub-surface layer	0.1
p_3	Power adjustment applied to topographic wetness index values for surface water	0.25
z_1	Assumed depth of the surface layer (cm)	5
Z_2	Assumed depth of the sub-surface layer (cm)	95
scn	Soil curve runoff number	0.82
cover	Fractional of vegetation cover	0.8*
R	Ratio of root water uptake from top layer relative to bottom layer	0.5
T	number of seconds in each time step of model run	86400
n_2	Pore size distribution parameter for controlling ground-water seepage	1.1
K_2	hydraulic conductivity parameter(s) for controlling ground-water seepage	0.5

*Assumed constant when running model at 100 m resolution. Estimated from aerial photographs and permitted to vary when running at one m resolution

To produce a gridded spatial dataset we used the observed historic dataset to derive the mean spatial anomaly for each month. These monthly anomalies were then spline interpolated to hourly and applied to the UKCP18 projected data. Functions and code for deriving sea-surface temperature data are included in the package 'UKCP18adjust' available from Github ('ilyamaclean/UKCP18adjust/').

Supplementary references

Kanamitsu M., Ebisuzaki W., Woollen J., Yang S.-K., Hnilo J., Fiorino M., Potter G. (2002). Ncep–doe amip-ii reanalysis (r-2). *Bulletin of the American Meteorological Society, 83*(11), 1631-1644.

Kasten F., Young A.T. (1989). Revised optical air mass tables and approximation formula. *Applied Optics, 28*(22), 4735-4738.

Maclean I.M.D, Mosedale J.R., Bennie J.J. (2019). Microclima: An r package for modelling meso-and microclimate. *Methods in Ecology and Evolution, 10*(2), 280-290.

Met Office (2018). *HadUK-Grid Gridded Climate Observations on a 1km grid over the UK for 1862-2017.* Available from: https://catalogue.ceda.ac.uk/.

Pebesma E., Graeler B.R (2019). Package 'gstat': Spatial and Spatio-Temporal Geostatistical Modelling, Prediction and Simulation. R package version 2.0.02.

Posselt R., Müller R., Trentmann J., Stockli R., Liniger M.A. (2014). A surface radiation climatology across two Meteosat satellite generations. *Remote Sensing of Environment, 142*, 103-110.

R Core Team (2018). *R: A language and environment for statistical computing. Version 3.5.1.*

Reynolds R.W., Smith T.M., Liu C., Chelton D.B., Casey K.S., Schlax M.G. (2007). Daily high-resolution-blended analyses for sea surface temperature. *Journal of Climate, 20*(22), 5473-5496.

Skartveit A., Olseth, J.A., Tuft, M.E. (1998). An hourly diffuse fraction model with correction for variability and surface albedo. *Solar Energy, 63*(3), 173-183.
