# Supplementary material for: Predicting future climate at high spatial and temporal resolution
Source: Glob Chang Biol. 2019 Nov 16;26(2):1003–11. doi: 10.1111/gcb.14876 (PMC7027457; doi:10.1111/gcb.14876)
Supplement: Supplementary file 2 [file GCB-26-1003-s002.pdf]

SUPPLEMENTARY RESULTS

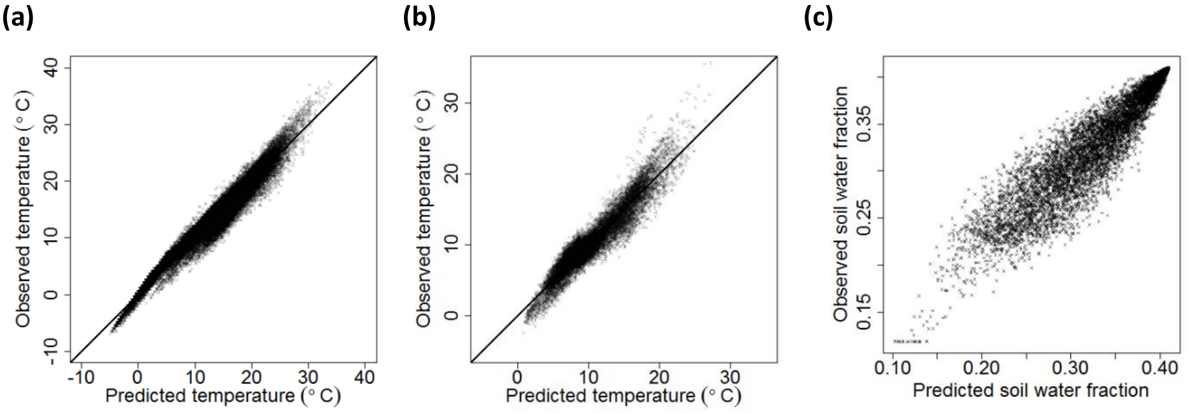

Fig. S5. Observed and predicted temperatures and soil water fractions. In (a) temperatures recorded by iButtons placed 1 m above the ground are compared to outputs obtained from the mesoclimate model, and in (b) temperatures recorded by iButtons 5 cm above-ground level are compared to the outputs of the microclimate model. In (c) 10,000 soil moisture measurements obtained from random locations across the study region using a Delta-T soil moisture probe are compared to soil moisture predictions in the upper soil layer.

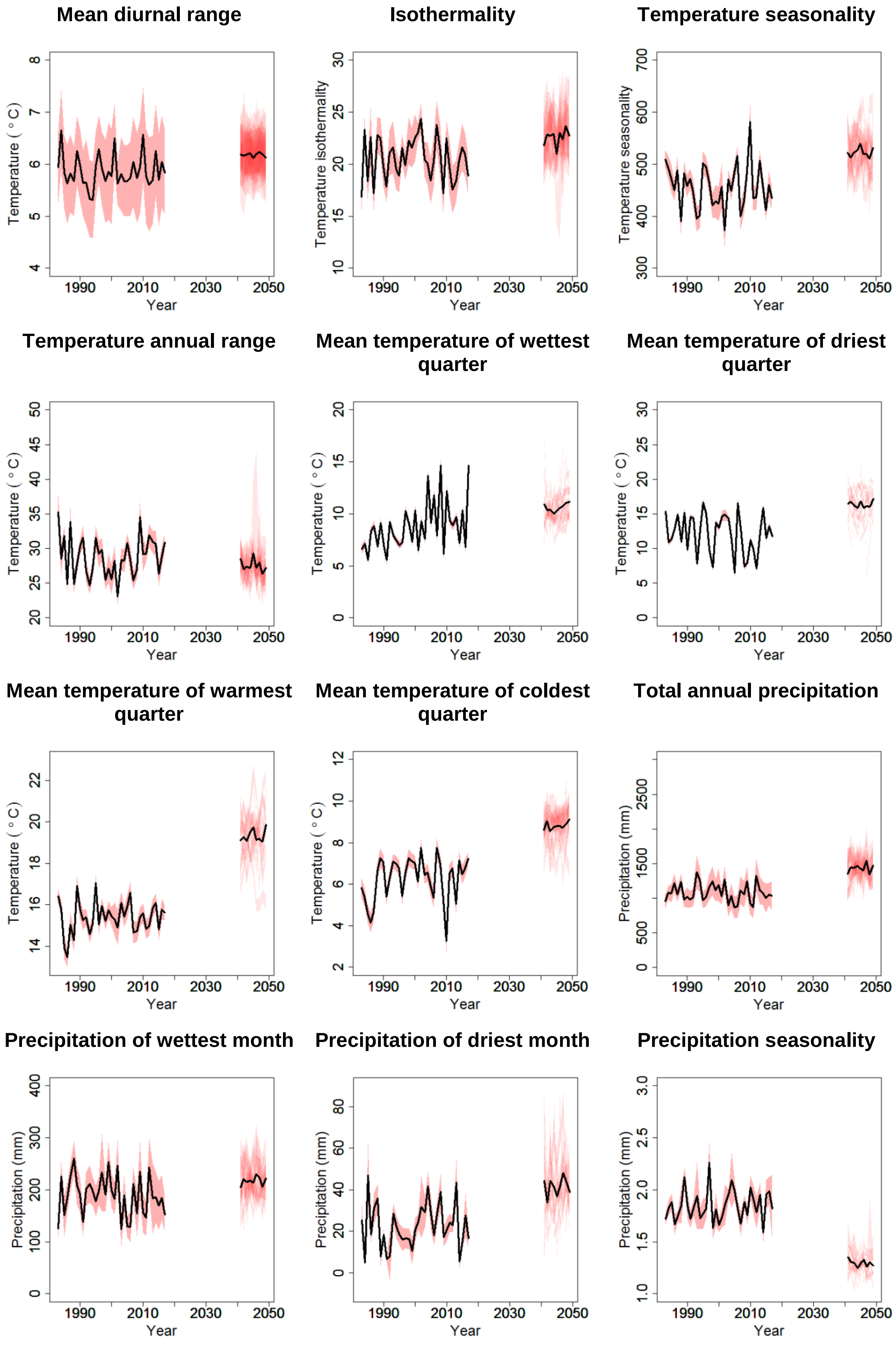

Mean diurnal range
Isothermality
Temperature seasonality
Temperature annual range
Mean temperature of wettest quarter
Mean temperature of driest quarter
Mean temperature of warmest quarter
Mean temperature of coldest quarter
Total annual precipitation
Precipitation of wettest month
Precipitation of driest month
Precipitation seasonality
Temperature (° C)
Temperature isothermality
Temperature seasonality
Precipitation (mm)
Year
1990
2010
2030
2050

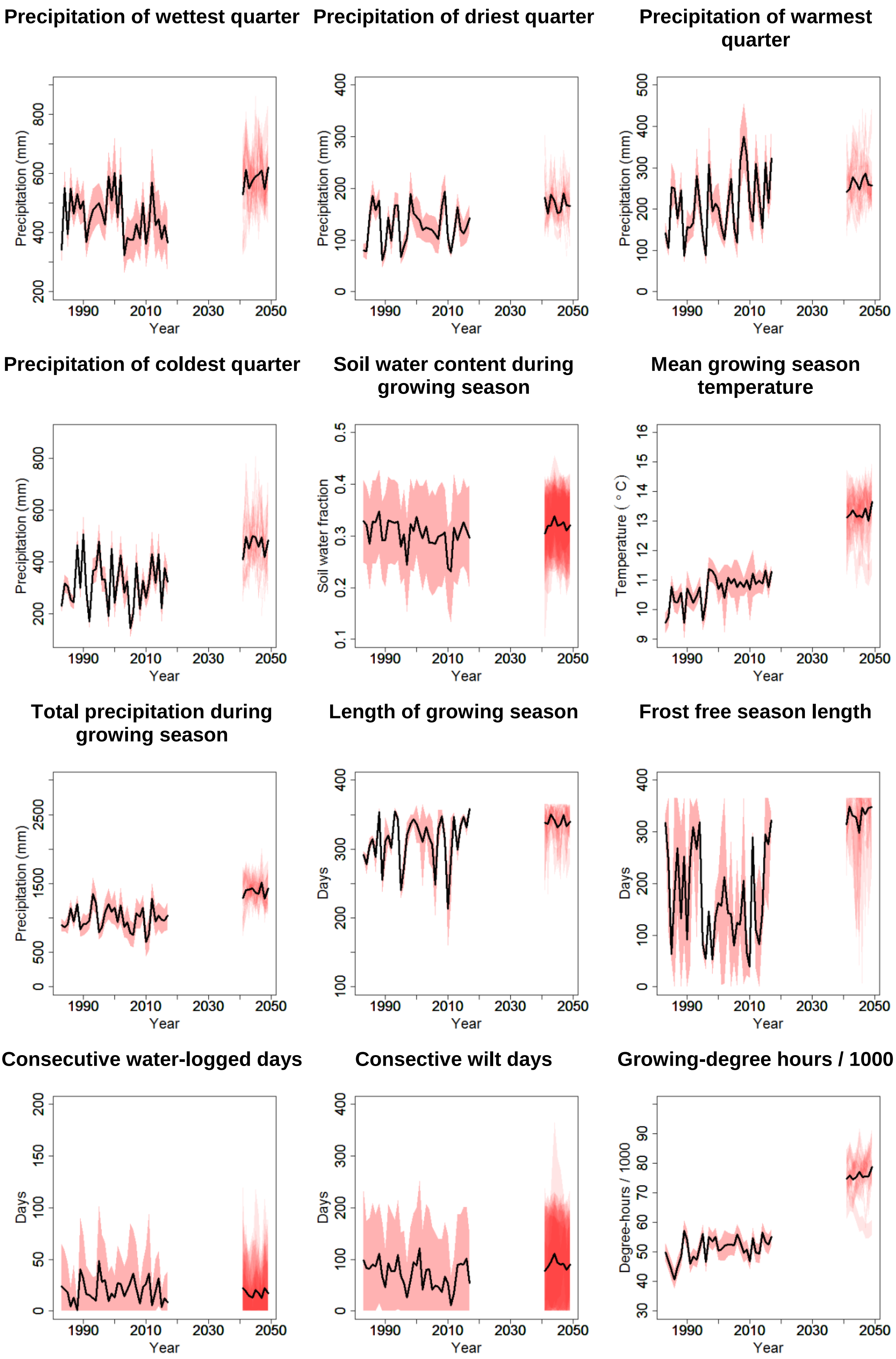
Precipitation of wettest quarter
Precipitation of driest quarter
Precipitation of warmest quarter
Precipitation of coldest quarter
Soil water content during growing season
Mean growing season temperature
Total precipitation during growing season
Length of growing season
Frost free season length
Consecutive water-logged days
Consective wilt days
Growing-degree hours / 1000
Precipitation (mm)
Soil water fraction
Temperature (° C)
Days
Degree-hours / 1000
Year
1990
2010
2030
2050

Fig. S6. Trends in bioclimate variables for the Lizard Peninsula under closed canopy. Black lines show the mean value across the study period and, in 2041-9 across model runs in each year. Pink shading in the 1983-2017 period represents ± 2 standard deviations in the spatial variability. In the 2041-2049 period, semi-transparent shading is used to depict ± 2 standard deviations in spatial variability of each model run and darker shading thus indicates greater overlap between model runs. More detailed variable descriptors are provided in Table 1. Trend plots for all variables in supplementary results.

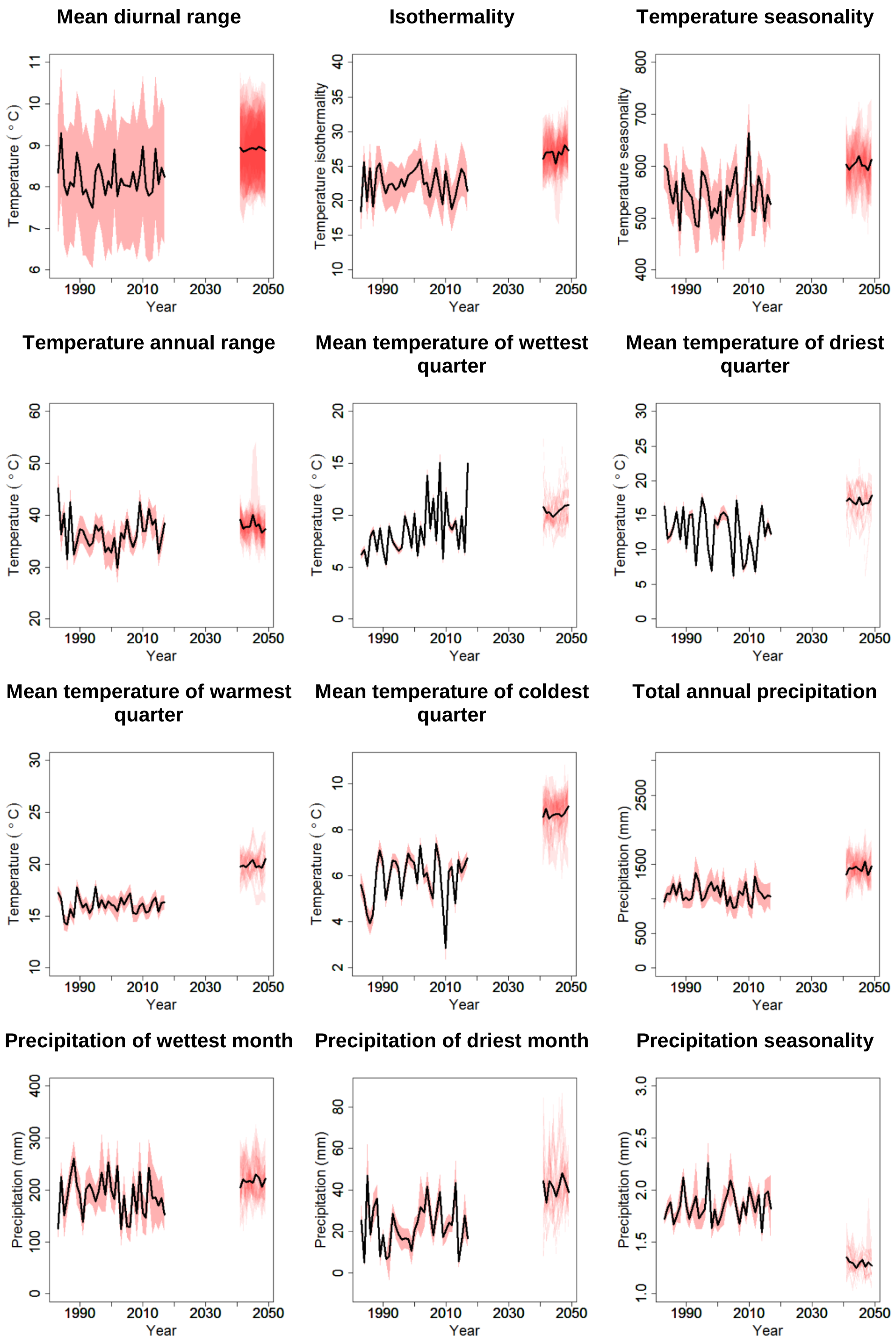

Mean diurnal range
Isothermality
Temperature seasonality
Temperature annual range
Mean temperature of wettest quarter
Mean temperature of driest quarter
Mean temperature of warmest quarter
Mean temperature of coldest quarter
Total annual precipitation
Precipitation of wettest month
Precipitation of driest month
Precipitation seasonality
Temperature (° C)
Temperature isothermality
Temperature seasonality
Precipitation (mm)
Year
1990
2010
2030
2050

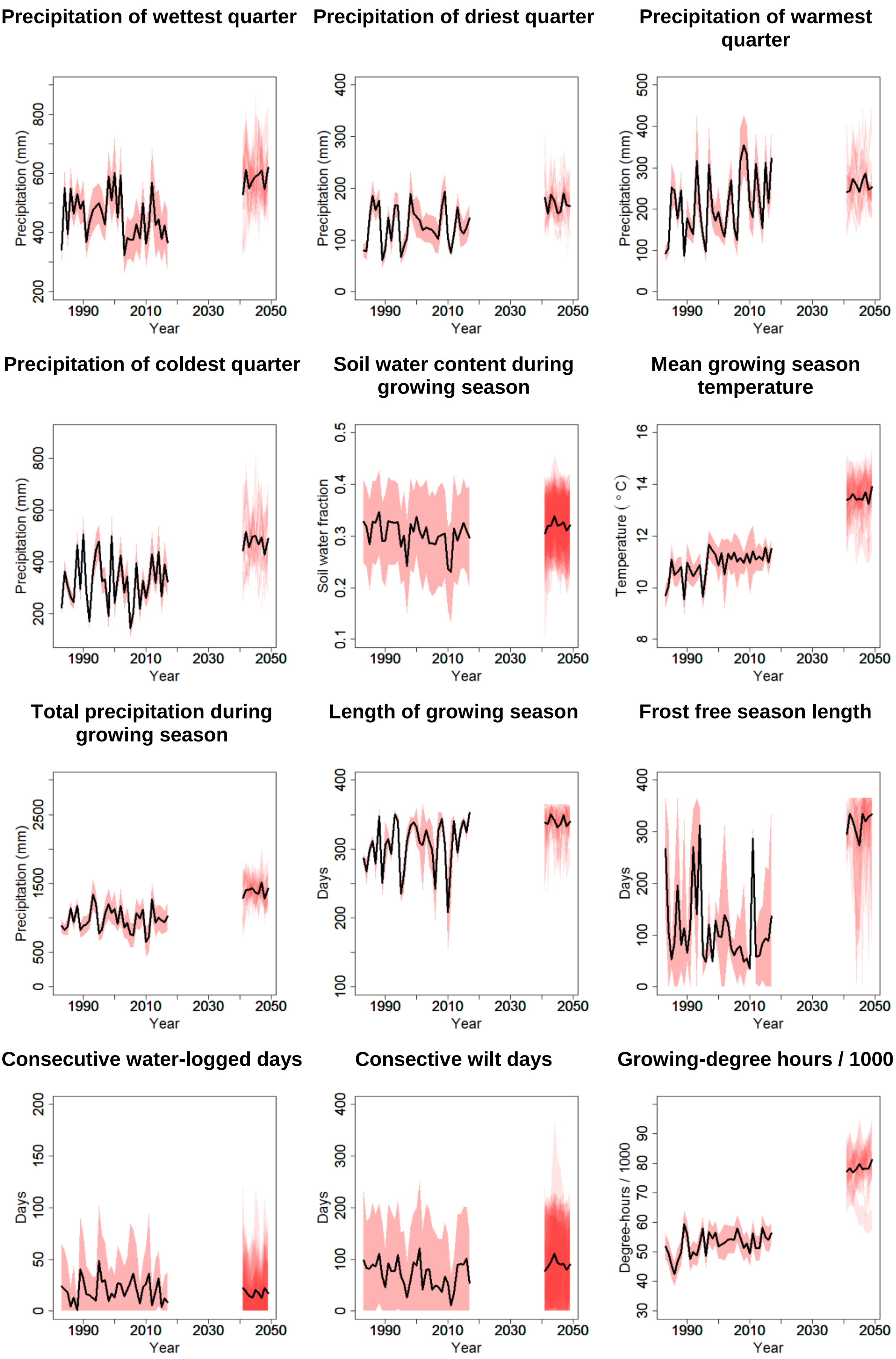
Precipitation of wettest quarter
Precipitation of driest quarter
Precipitation of warmest quarter
Precipitation of coldest quarter
Soil water content during growing season
Mean growing season temperature
Total precipitation during growing season
Length of growing season
Frost free season length
Consecutive water-logged days
Consective wilt days
Growing-degree hours / 1000
Precipitation (mm)
Soil water fraction
Temperature (° C)
Days
Degree-hours / 1000
Year
1990
2010
2030
2050

Fig. S7. Trends in bioclimate variables for the Lizard Peninsula under open canopy. Black lines show the mean value across the study period and, in 2041-9 across model runs in each year. Pink shading in the 1983-2017 period represents ± 2 standard deviations in the spatial variability. In the 2041-2049 period, semi-transparent shading is used to depict ± 2 standard deviations in spatial variability of each model run and darker shading thus indicates greater overlap between model runs. More detailed variable descriptors are provided in Table 1. Trend plots for all variables in supplementary results.

Mean diurnal range

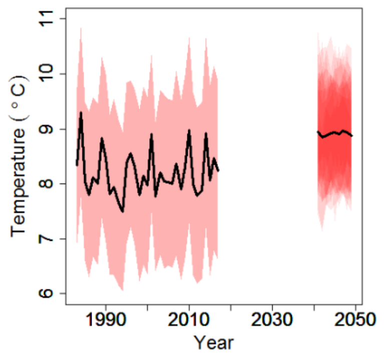

Isothermality

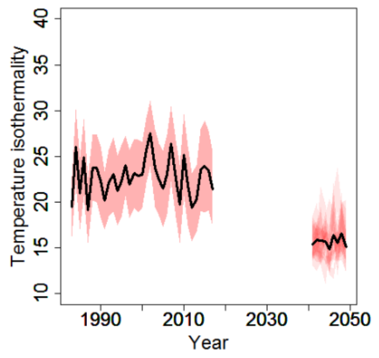

Temperature seasonality

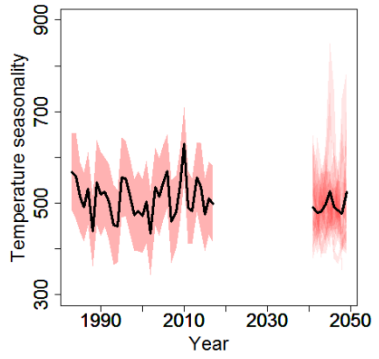

Temperature annual range

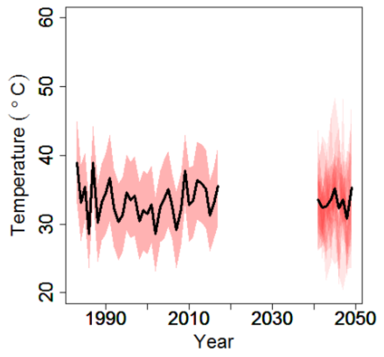

Mean temperature of wettest quarter

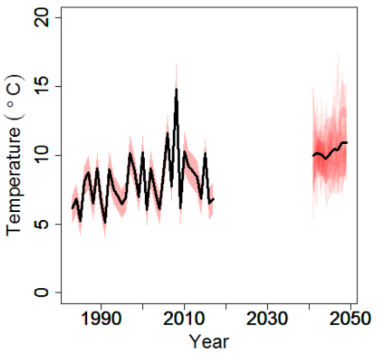

Mean temperature of driest quarter

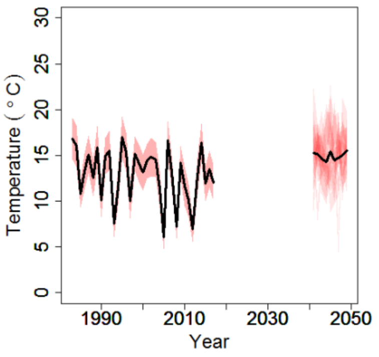

Mean temperature of warmest quarter

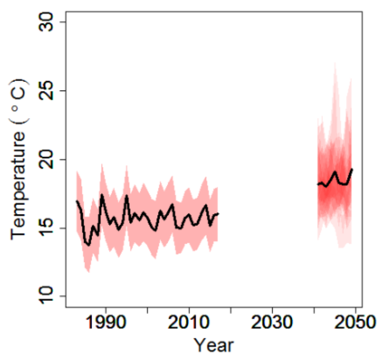

Mean temperature of coldest quarter

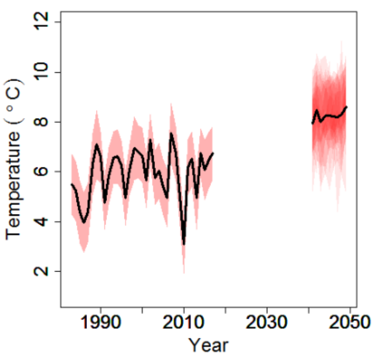

Total annual precipitation

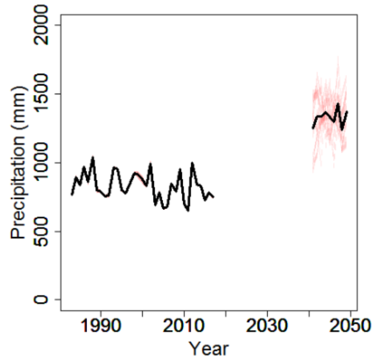

Precipitation of wettest month

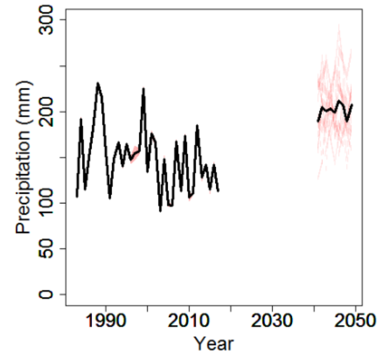

Precipitation of driest month

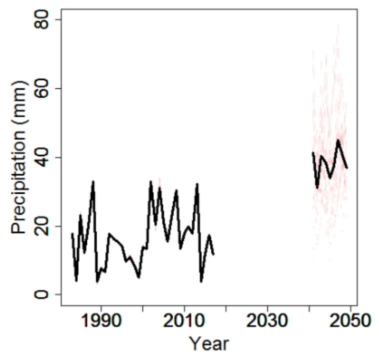

Precipitation seasonality

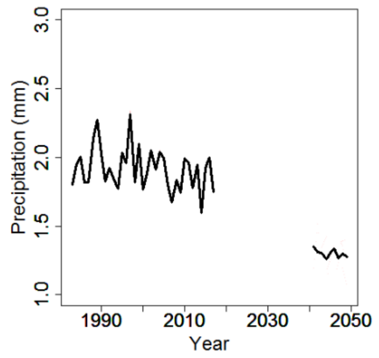

Precipitation of wettest quarter

Precipitation of driest quarter

Precipitation of warmest quarter

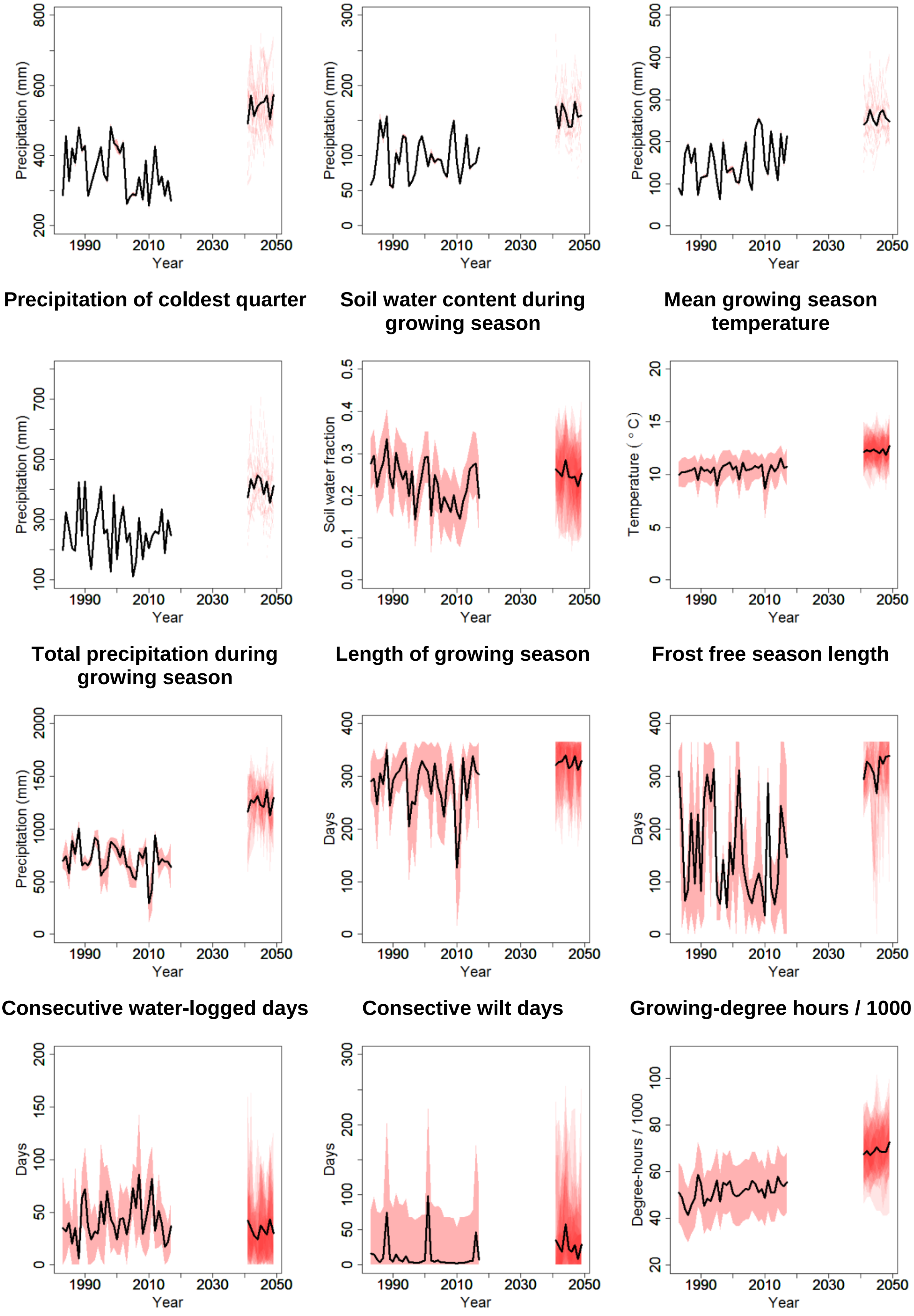
Precipitation (mm)
Year
Precipitation of coldest quarter
Soil water content during growing season
Mean growing season temperature
Soil water fraction
Temperature (° C)
Total precipitation during growing season
Length of growing season
Frost free season length
Days
Consecutive water-logged days
Consective wilt days
Growing-degree hours / 1000
Degree-hours / 1000

Fig. S8. Trends in bioclimate variables for Caerthillean Cove. Black lines show the mean value across the study period and, in 2041-9 across model runs in each year. Pink shading in the 1983-2017 period represents ± 2 standard deviations in the spatial variability. In the 2041-2049 period, semi-transparent shading is used to depict ± 2 standard deviations in spatial variability of each model run and darker shading thus indicates greater overlap between model runs. More detailed variable descriptors are provided in Table 1. Trend plots for all variables in supplementary results.

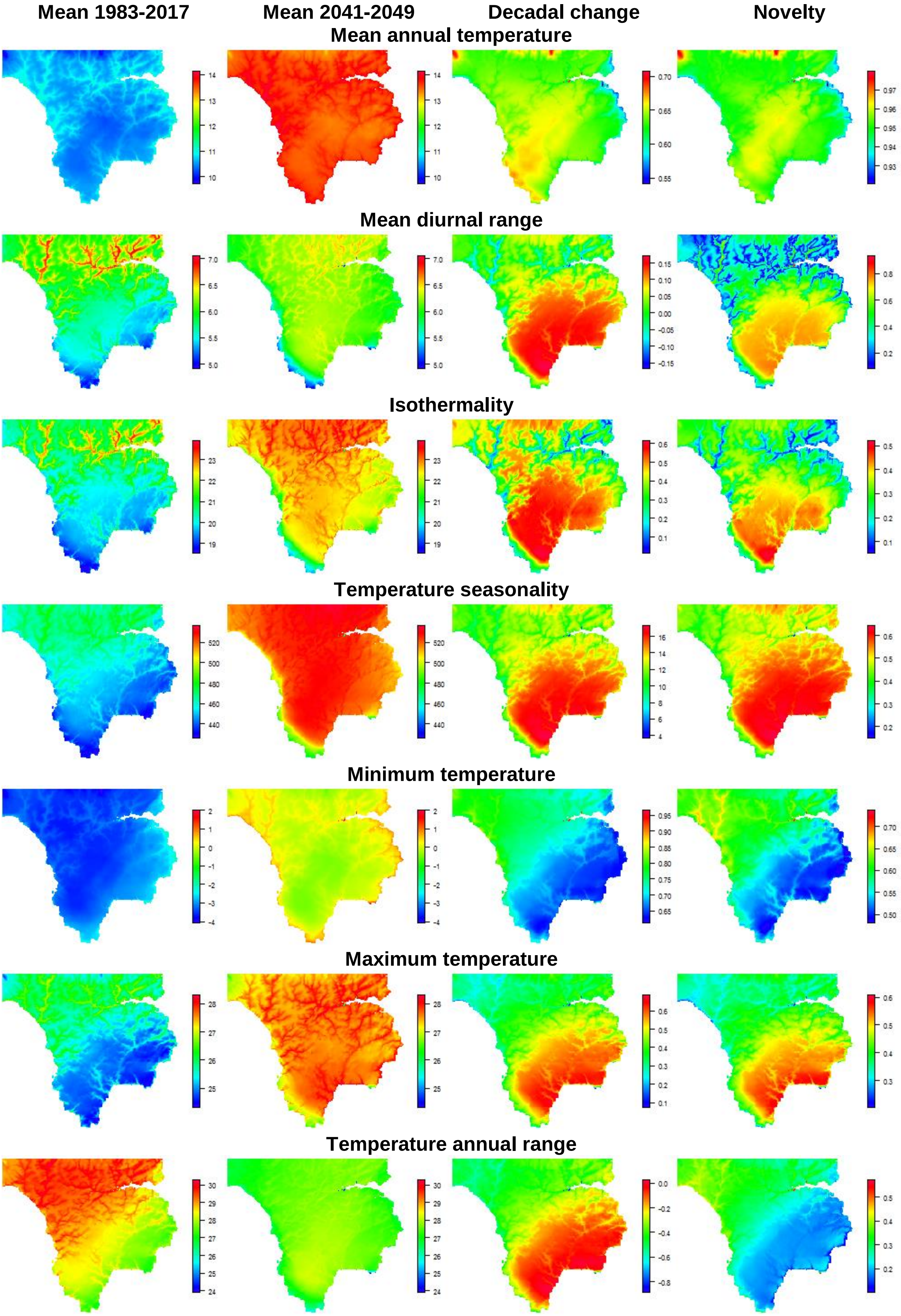
Mean 1983-2017
Mean 2041-2049
Decadal change
Novelty
Mean annual temperature
Mean diurnal range
Isothermality
Temperature seasonality
Minimum temperature
Maximum temperature
Temperature annual range

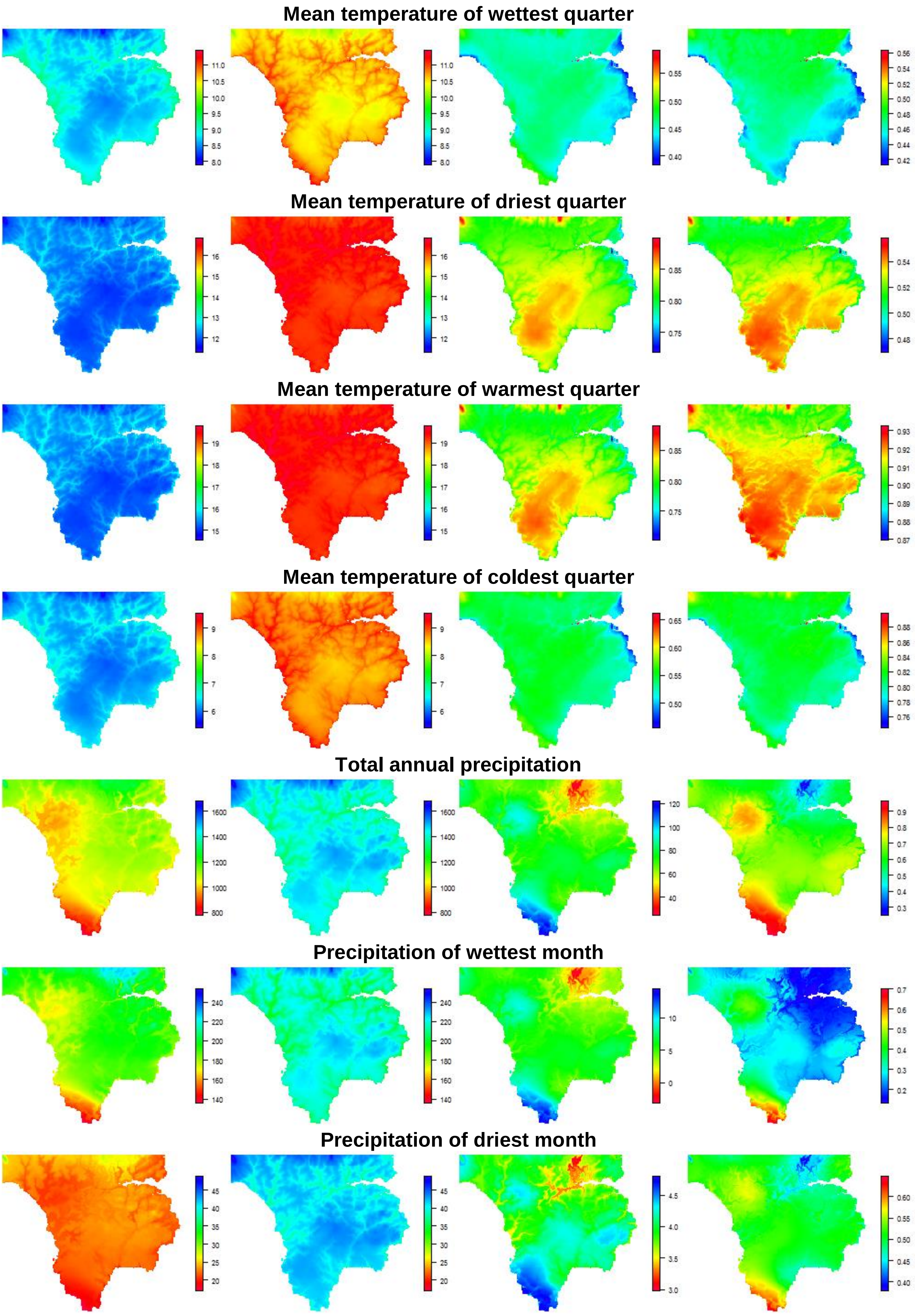
Mean temperature of wettest quarter
Mean temperature of driest quarter
Mean temperature of warmest quarter
Mean temperature of coldest quarter
Total annual precipitation
Precipitation of wettest month
Precipitation of driest month

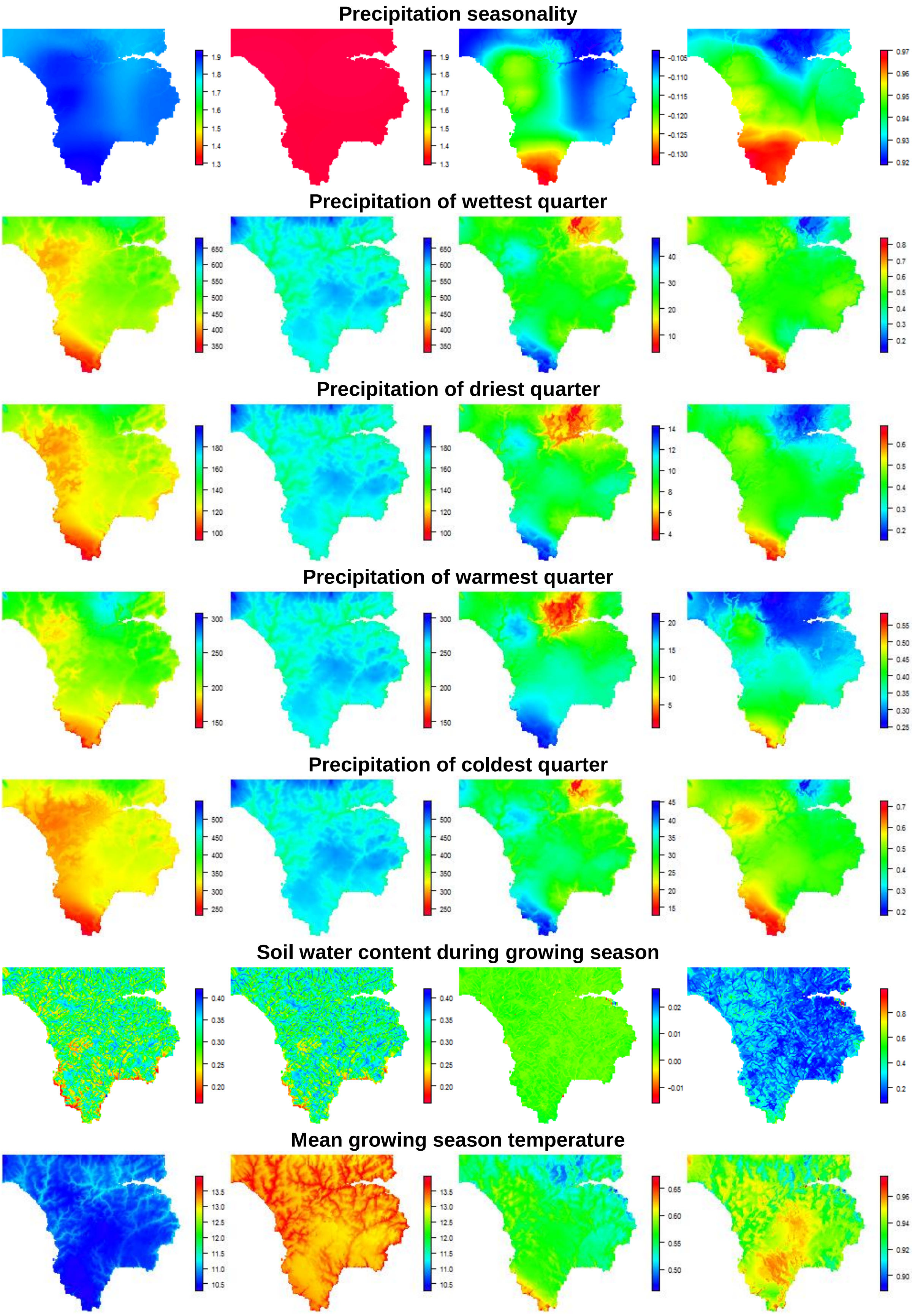
Precipitation seasonality
Precipitation of wettest quarter
Precipitation of driest quarter
Precipitation of warmest quarter
Precipitation of coldest quarter
Soil water content during growing season
Mean growing season temperature

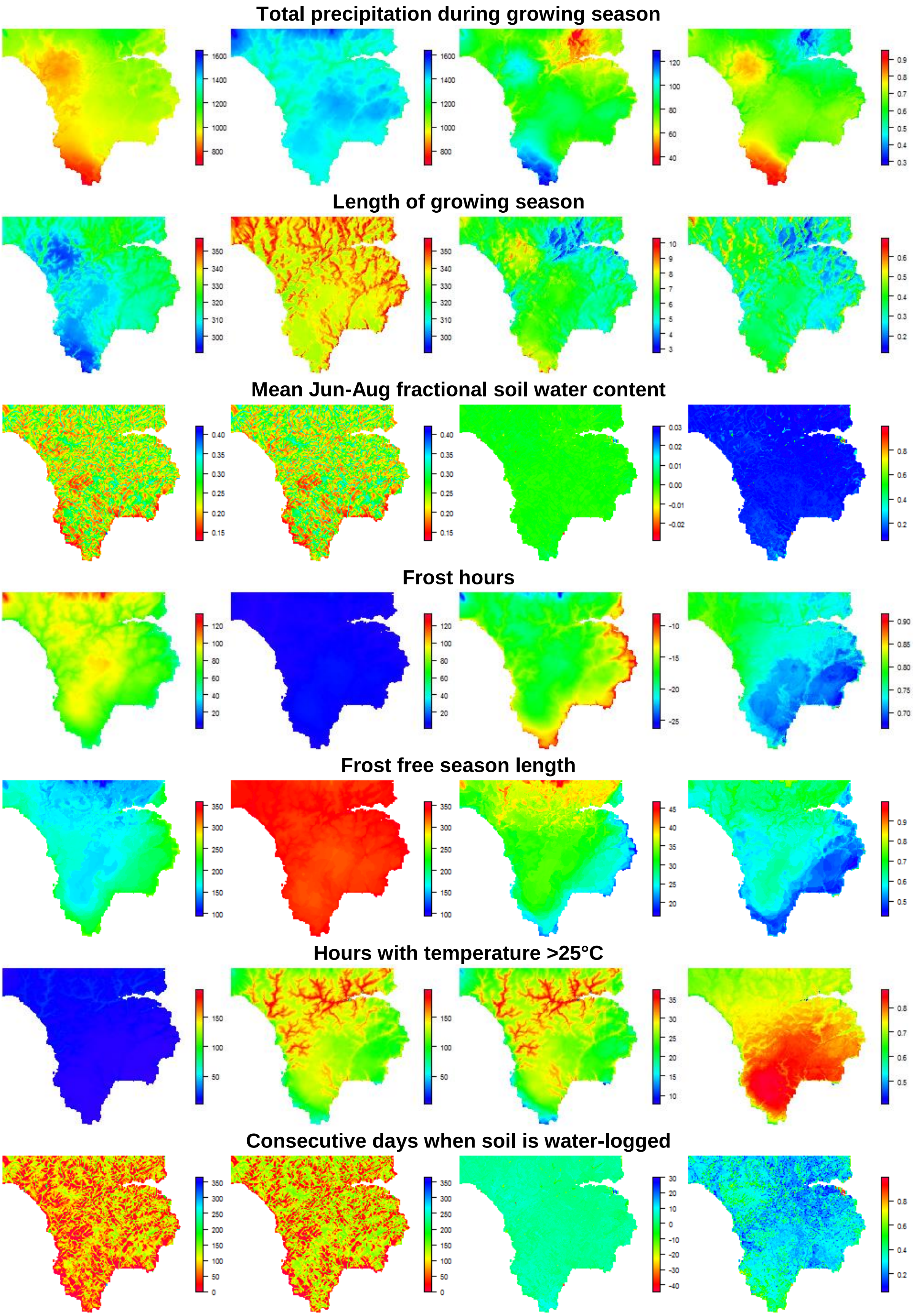
Total precipitation during growing season
Length of growing season
Mean Jun-Aug fractional soil water content
Frost hours
Frost free season length
Hours with temperature >25°C
Consecutive days when soil is water-logged

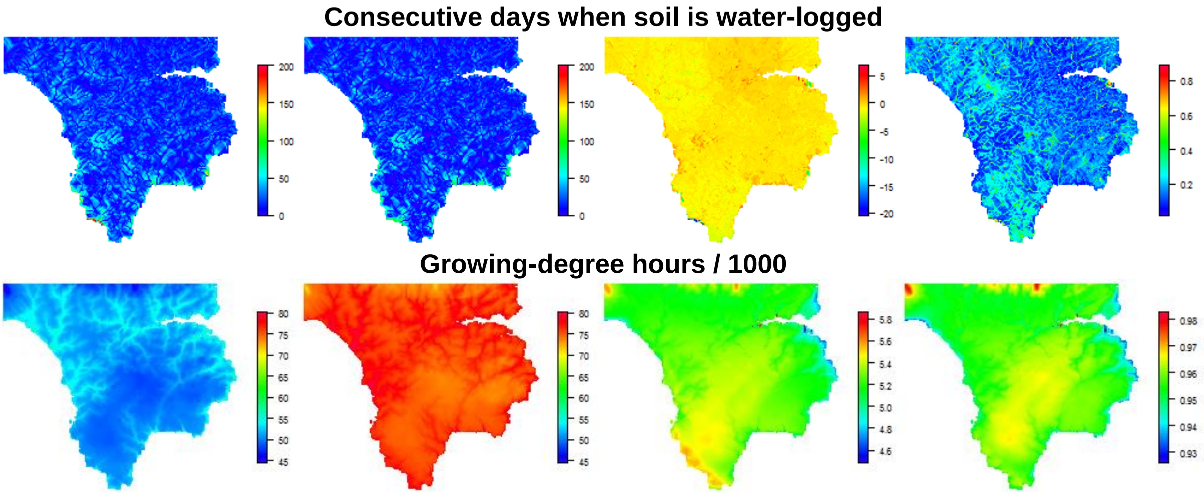

Fig. S9. Maps of bioclimate variables under closed canopy. Decadal changes were derived using linear regression on yearly values. Novelty represents the proportional overlap in the frequency distribution of annual values in 1983-2017 with that of annual values for each model run in 2041-2049 (0 = complete overlap, 1 = no overlap).

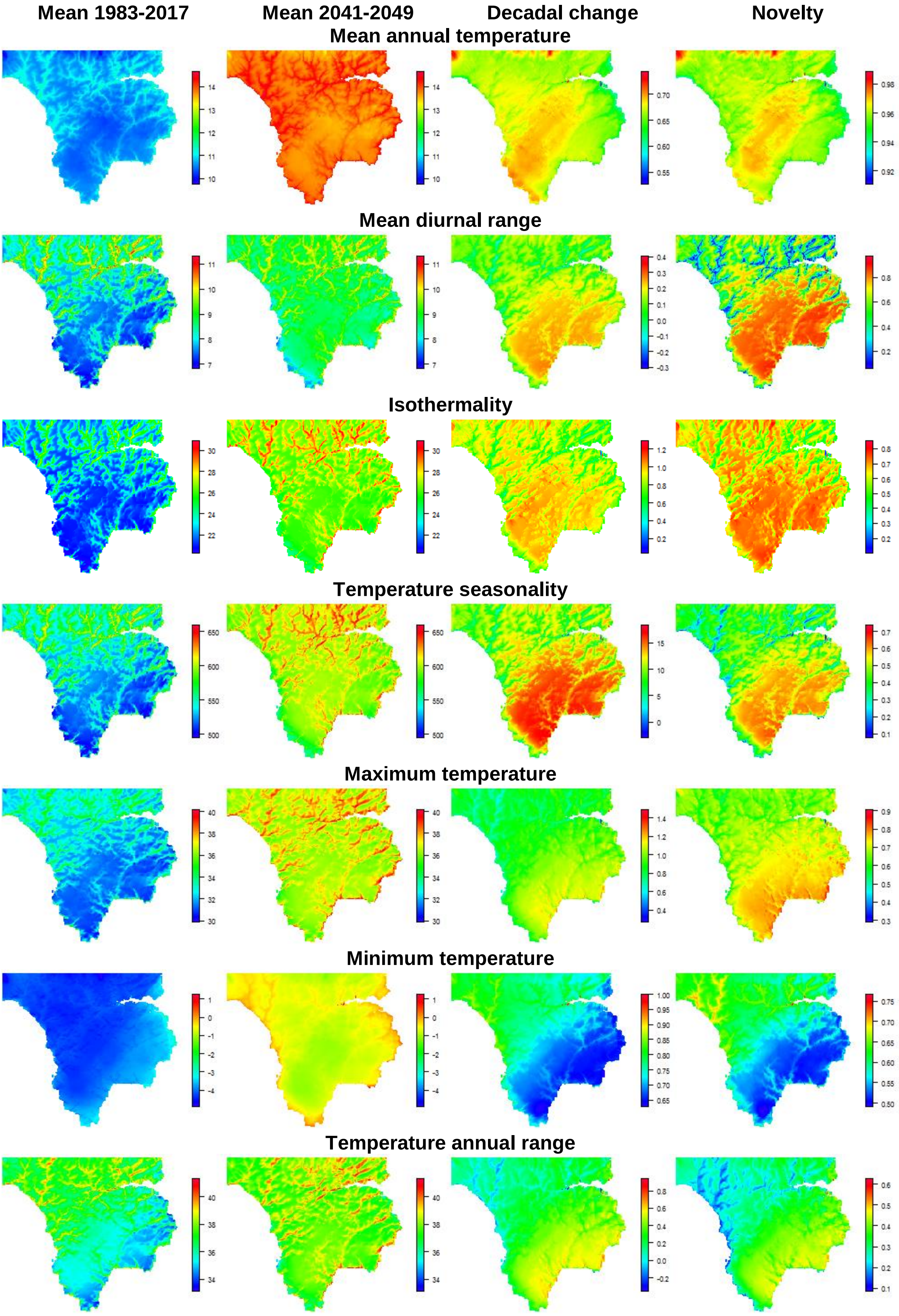
Mean 1983-2017
Mean 2041-2049
Decadal change
Novelty
Mean annual temperature
Mean diurnal range
Isothermality
Temperature seasonality
Maximum temperature
Minimum temperature
Temperature annual range

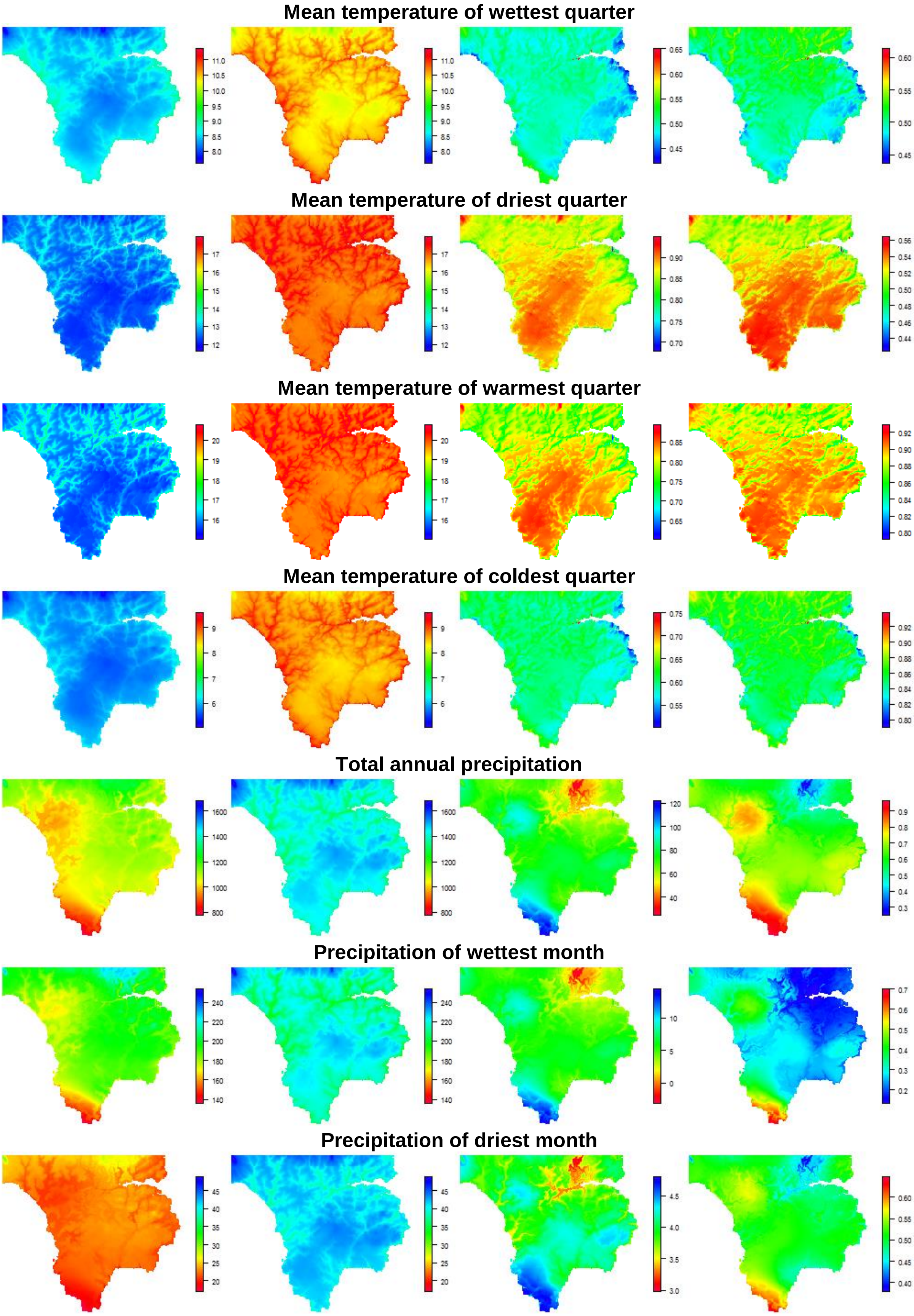
Mean temperature of wettest quarter
Mean temperature of driest quarter
Mean temperature of warmest quarter
Mean temperature of coldest quarter
Total annual precipitation
Precipitation of wettest month
Precipitation of driest month

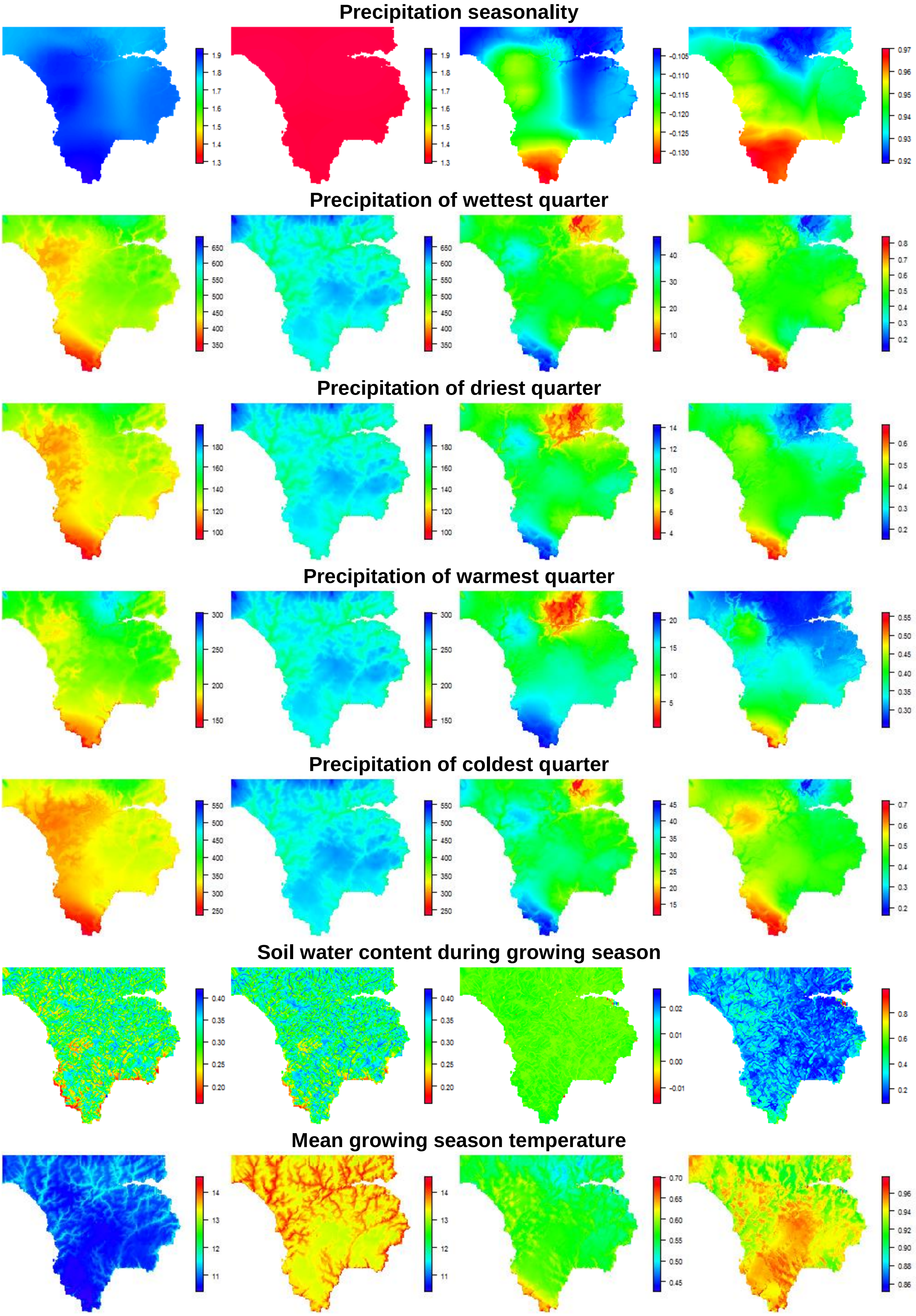
Precipitation seasonality
Precipitation of wettest quarter
Precipitation of driest quarter
Precipitation of warmest quarter
Precipitation of coldest quarter
Soil water content during growing season
Mean growing season temperature

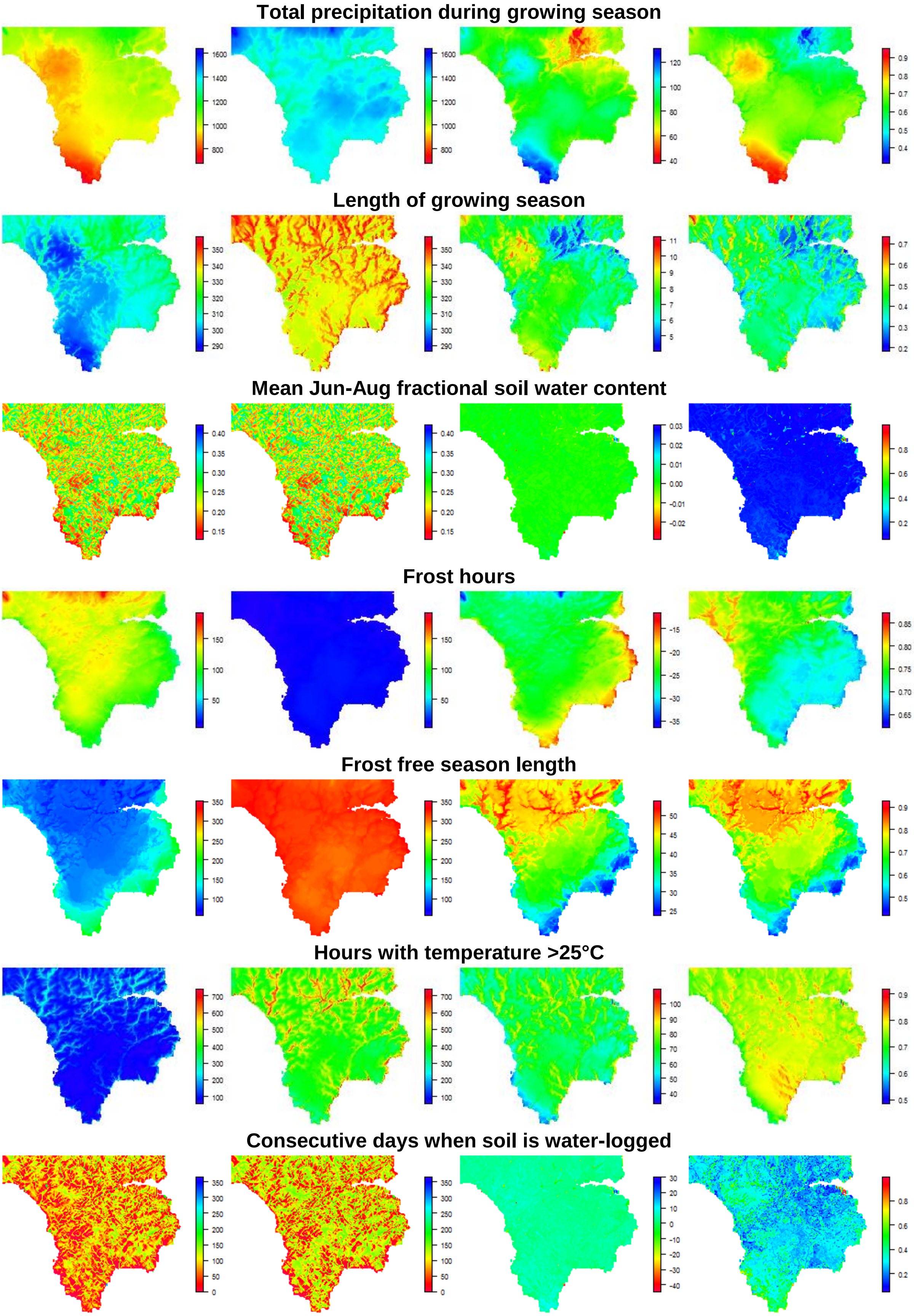
Total precipitation during growing season
Length of growing season
Mean Jun-Aug fractional soil water content
Frost hours
Frost free season length
Hours with temperature >25°C
Consecutive days when soil is water-logged

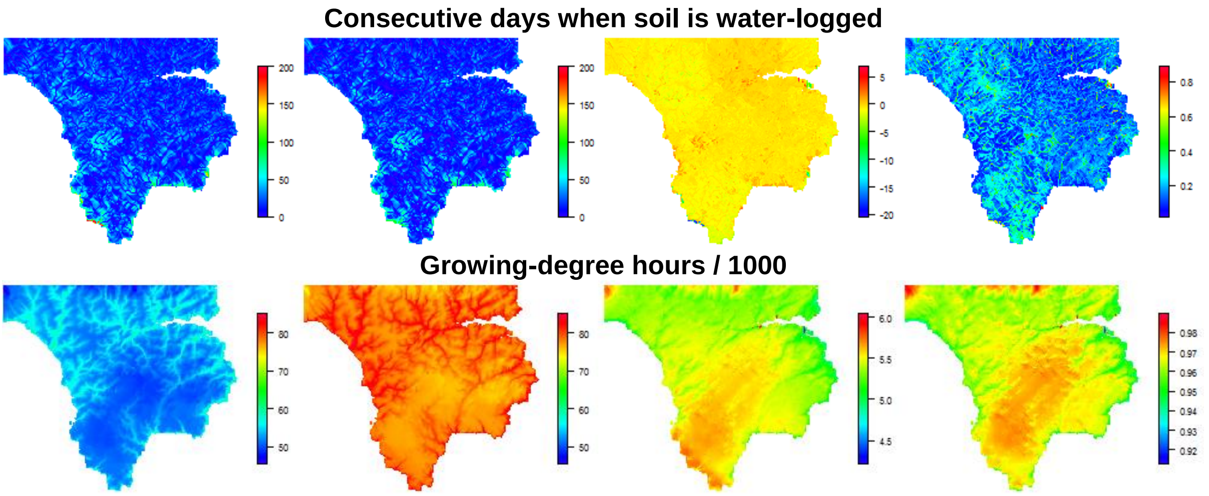

Fig. S10. Maps of selected bioclimate variables under open canopy. Decadal changes were derived using linear regression on yearly values. Novelty represents the proportional overlap in the frequency distribution of annual values in 1983-2017 with that of annual values for each model run in 2041-2049 (0 = complete overlap, 1 = no overlap).

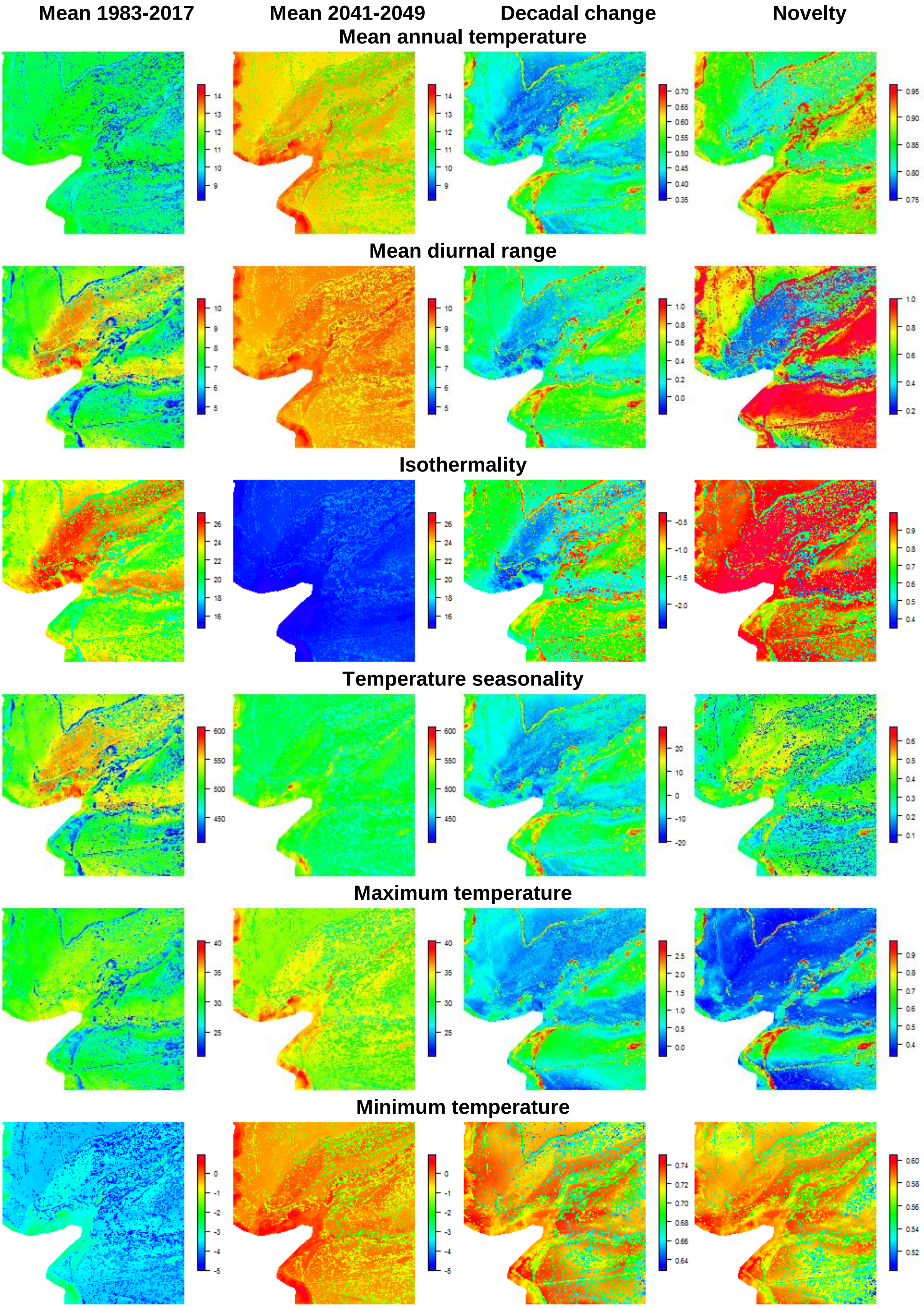
Mean 1983-2017
Mean 2041-2049
Decadal change
Novelty
Mean annual temperature
Mean diurnal range
Isothermality
Temperature seasonality
Maximum temperature
Minimum temperature

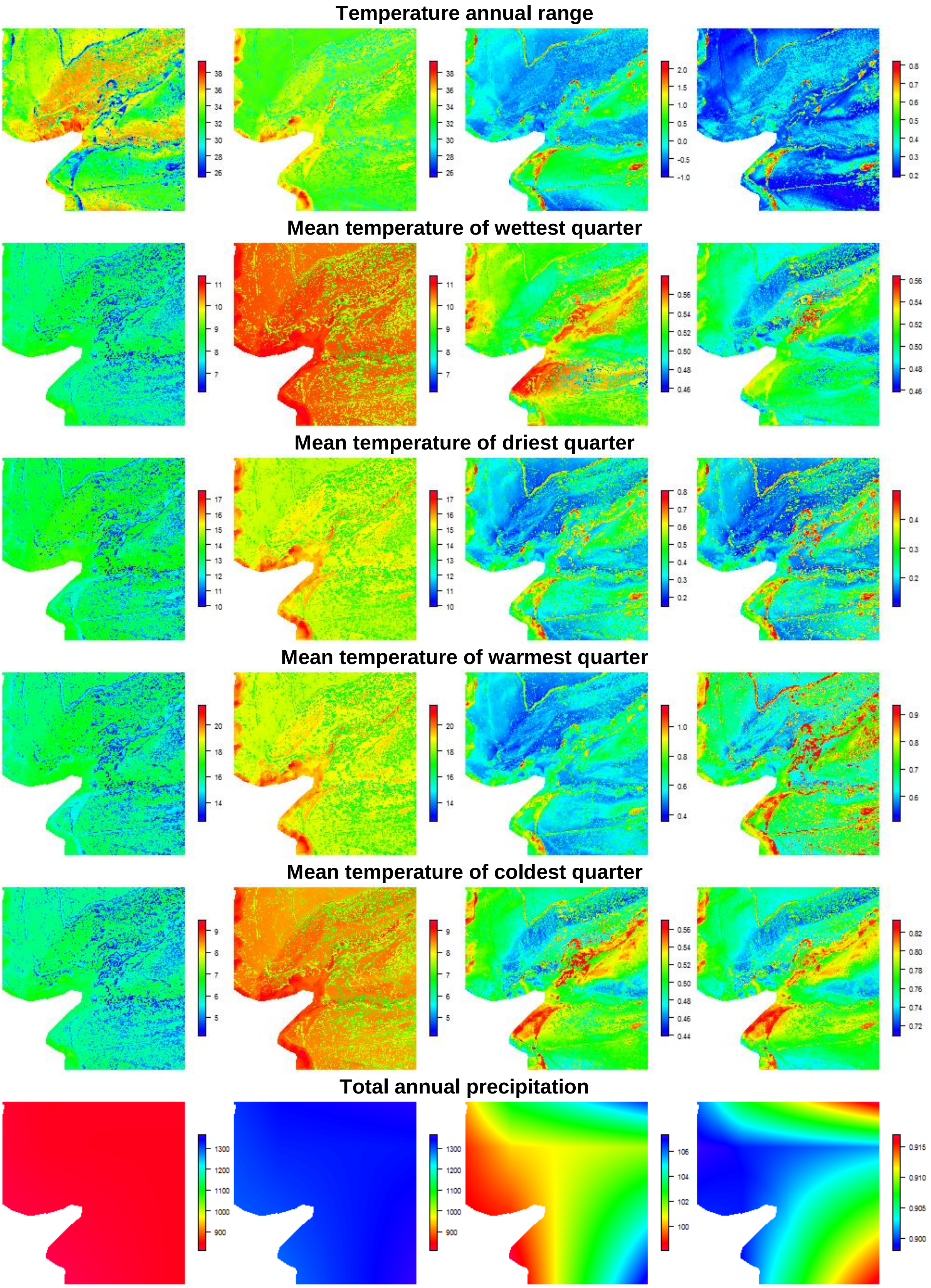
Temperature annual range
Mean temperature of wettest quarter
Mean temperature of driest quarter
Mean temperature of warmest quarter
Mean temperature of coldest quarter
Total annual precipitation

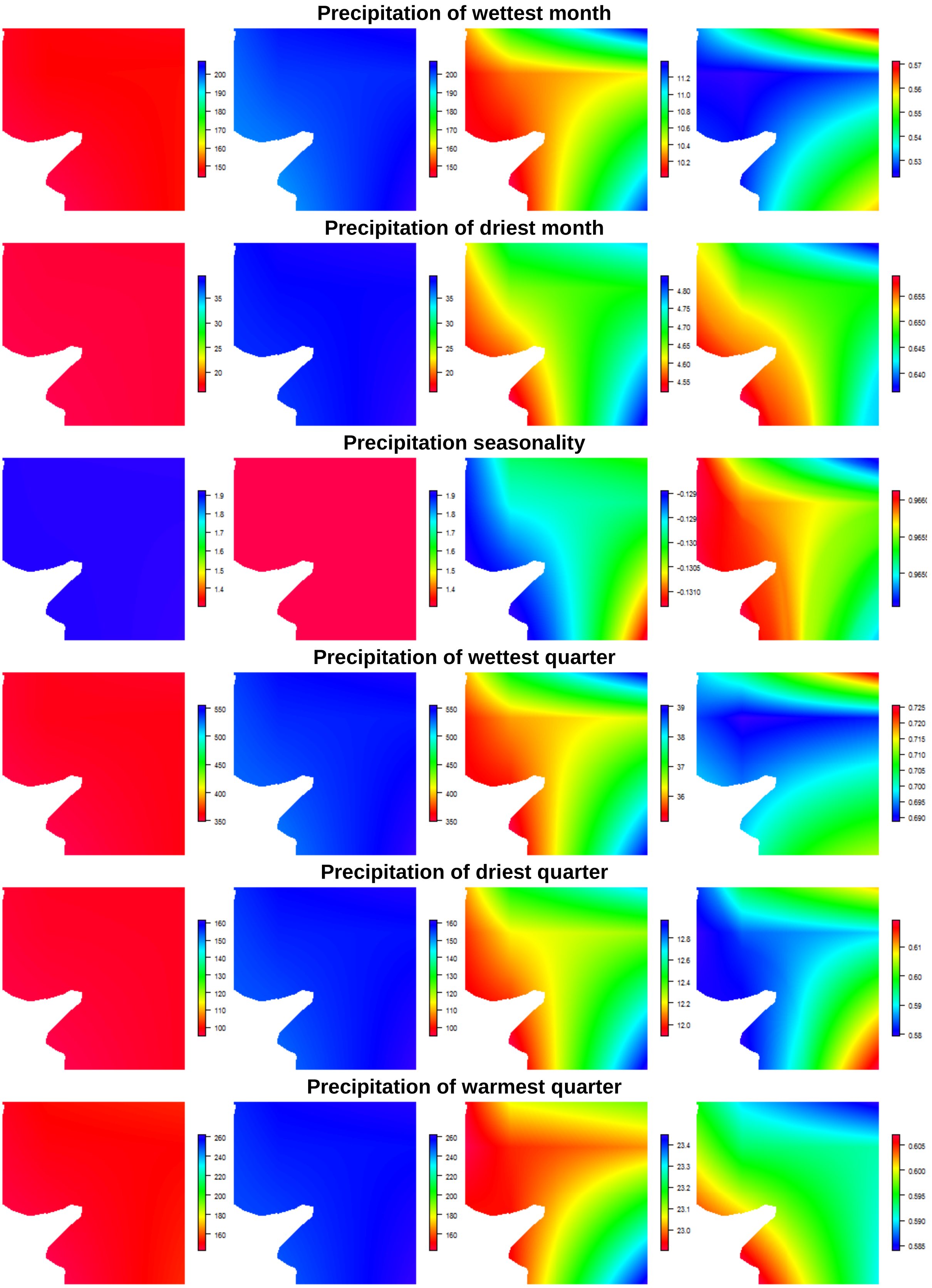
Precipitation of wettest month
Precipitation of driest month
Precipitation seasonality
Precipitation of wettest quarter
Precipitation of driest quarter
Precipitation of warmest quarter

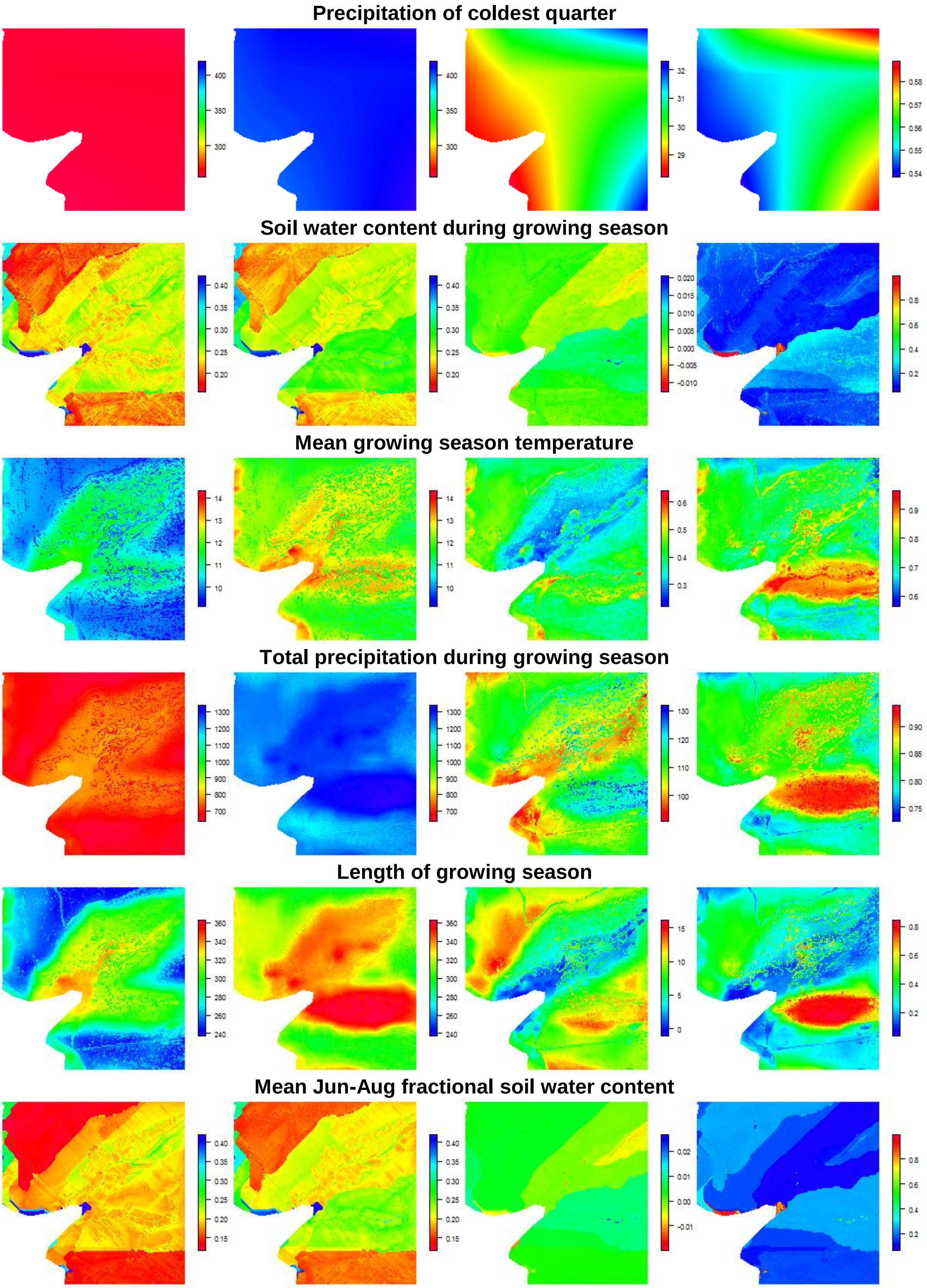
Precipitation of coldest quarter
Soil water content during growing season
Mean growing season temperature
Total precipitation during growing season
Length of growing season
Mean Jun-Aug fractional soil water content

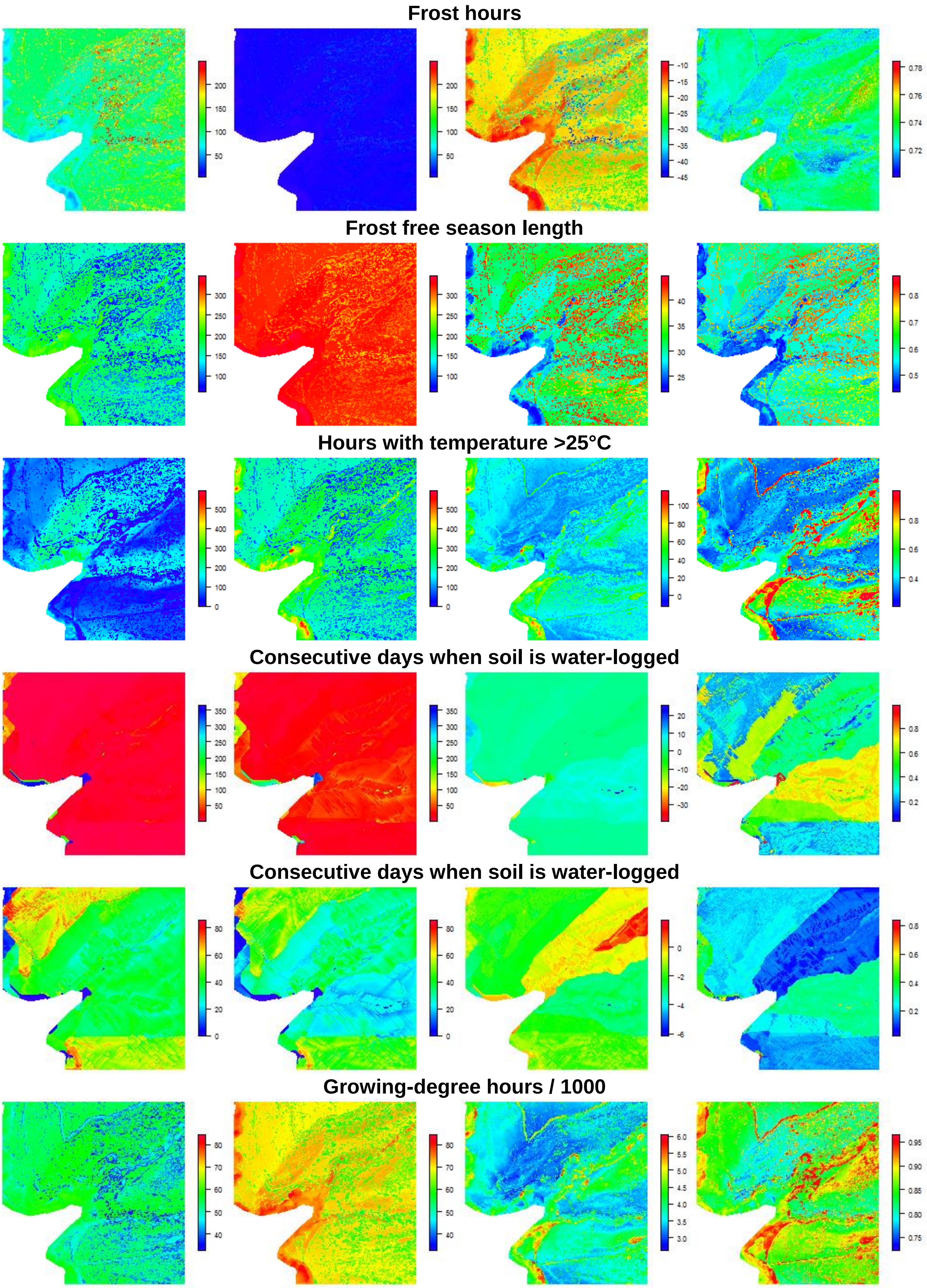

Fig. S11. Maps of selected bioclimate variables in Caerthillean Cove. Decadal changes were derived using linear regression on yearly values. Novelty represents the proportional overlap in the frequency distribution of annual values in 1983-2017 with that of annual values for each model run in 2041-2049 (0 = complete overlap, 1 = no overlap).
